# Advanced methods and novel biomarkers in autoimmune diseases ‑ a review of the recent years progress in systemic lupus erythematosus

**DOI:** 10.3389/fmed.2023.1183535

**Published:** 2023-06-23

**Authors:** Kristin Andreassen Fenton, Hege Lynum Pedersen

**Affiliations:** ^1^UiT The Arctic University of Norway, Tromsø, Norway; ^2^Centre of Clinical Research and Education, University Hospital of North Norway, Tromsø, Norway

**Keywords:** SLE, systemic lupus erythematosus, transcriptomics (RNA-Seq), PET/MRI, imaging - computed tomography, organ specific, urine biomarker, biomarker

## Abstract

There are several autoimmune and rheumatic diseases affecting different organs of the human body. Multiple sclerosis (MS) mainly affects brain, rheumatoid arthritis (RA) mainly affects joints, Type 1 diabetes (T1D) mainly affects pancreas, Sjogren’s syndrome (SS) mainly affects salivary glands, while systemic lupus erythematosus (SLE) affects almost every organ of the body. Autoimmune diseases are characterized by production of autoantibodies, activation of immune cells, increased expression of pro-inflammatory cytokines, and activation of type I interferons. Despite improvements in treatments and diagnostic tools, the time it takes for the patients to be diagnosed is too long, and the main treatment for these diseases is still non-specific anti-inflammatory drugs. Thus, there is an urgent need for better biomarkers, as well as tailored, personalized treatment. This review focus on SLE and the organs affected in this disease. We have used the results from various rheumatic and autoimmune diseases and the organs involved with an aim to identify advanced methods and possible biomarkers to be utilized in the diagnosis of SLE, disease monitoring, and response to treatment.

## Introduction

Systemic chronic inflammation (SCI) is the presence of a slow low-grade inflammation lasting for longer periods from several months to years (1). It is caused by the constant activation and infiltration of immune cells into the affected organs. SCI may advance into autoimmune diseases with the characteristic development of autoantigen-specific lymphocytes and autoantibodies that may progress into clinical disease manifestations (2). However, because the preclinical phase often is silent, it is difficult to predict the progression from harmless inflammation to activation of autoantigen-specific T and B cells leading to production of autoantibodies and activation of effector cells, the hallmarks of autoimmune diseases. There is still a lack of detailed clinical and molecular knowledge that can lead to a better understanding of the pathogenesis behind such diseases, and there is a need for suitable predictive biomarkers that can identify patients that are at risk to develop autoimmune diseases (2).

Systemic lupus erythematosus (SLE) is a chronic inflammatory autoimmune disease disposed to inflammation in nearly all organs. The production of autoantibodies (specifically anti-double stranded (ds) DNA) and the formation of immune complexes is a hallmark of SLE (3), but there is still a lack of clinically useful diagnostic markers and markers that can predict organ involvement.

Biomarkers are as the word imply, biological markers. There are different kinds of biomarkers such as diagnostic, mechanistic, clinical, therapeutic. These markers can identify gene variants, gene or protein expression, disease monitoring, and therapeutic response. Common for all, is that they objectively predict biological characteristics that is difficult to observe. Perhaps the biggest advantage of implementing good, clinical biomarkers could be the implementation of precision medicine. In a review article by Giacomelli et al. (4) regarding biomarkers in autoimmune rheumatic diseases, they concluded that biomarkers and personalized medicine (e.g., precision medicine that target treatment to individual patients based on precise and specific information of the patients disease) would be future central points in management of patients affected by rheumatic and autoimmune diseases.

Transcriptomics is the study of expression profiling of levels of mRNA in an organism at a given time. RNA sequencing (RNAseq) allows the characterization of different states of cells or tissue by expression patterns. Utilizing these methods, the molecular mechanisms underlying a phenotype and identifying differential expressed biomarkers in diseases compared to healthy individuals, can be investigated. The use of transcriptomics in autoimmune diseases have increased our knowledge of the pathophysiology of such diseases. In particular, the immune cells, signaling pathways, and the genes involved have been in focus (5). Monocytes and macrophages, including tissue specific resident macrophages, are key cells commonly expressed across different inflammatory and autoimmune diseases. However, the biggest challenge still lies in linking the results from RNA-seq into clinical application.

One of the newest techniques in transcriptomic analyses, single cell RNA sequencing (scRNAseq), was developed in 2009 by Tang et al. and has been one of the most used transcriptomic methods the last decade (6). In autoimmune diseases, the use of scRNAseq has opened for the identification of cell and molecular biomarkers that may predict disease progression, disease outcome, and individualized therapy (7). The use of scRNAseq in autoimmune inflammatory rheumatic disease has recently been reviewed and highlight the current challenges to overcome before we can utilize novel findings in new advanced diagnostic tools (8). The single cell multiomics data complexity require interdisciplinary collaborations, the high cost of reagents and equipment prevents the profiling of large patients cohorts, and in the end, few user-friendly, easy accessible interface linking the results with the whole research field makes the transition into the clinics difficult (8).

Molecular imaging allows for the detection of cellular and molecular changes within living species. It can characterize and measure biological processes *in vivo* and allows for visualization of the whole body down to cellular resolution level (9). Positron emission tomography (PET) together with computed tomography (CT) (PET/CT) or magnetic resonance imaging (MRI) (PET/MRI) take advantage of tracers with radioactive isotopes that can measure metabolic activity in cells and organs. Carbohydrate metabolism [glucose, ^18^F fluorodeoxyglucose (FDG) and mannose receptor (^18^F-fluoro-D-mannose; ^18^F-FDM)], chemokine receptors (C-X-C Motif Chemokine Receptor 4 (CXCR4), ^68^Ga-pentixafor), somatostatin receptors (^68^Ga-labeled DOTA-peptides), cell adhesion molecules (CAMs, ^18^F-galacto-RGD, ^68^Ga-PRGD2, and ^18^F-fluciclatide), fibroblast Activation Protein-α (FAP, ^68^Ga-FAPI), folate receptor (FR, ^18^F-fluoro-PEG-folate), and mitochondrial translocator protein (TSPO, ^11^C-PK11195 or ^18^F-flutriciclamide) are some of the targets of tracers developed to detect inflammation in different diseases (10).

Given the fast development of new, sophisticated techniques generating vast amount of data in combination with machine learning and artificial intelligence (AI), there are tremendous opportunities ahead of us. The results should lead to new advancements in disease diagnosis, monitoring and response to therapy. Despite good research on animal models, there is a knowledge gap when it comes to new biomarkers, methods, and treatment regarding SLE. Previous work has mostly focused on cutaneous SLE and lupus nephritis (LN), but since SLE is a systemic disease, we wanted to review what is known about SLE and organs such as joint, liver, pancreas, brain, salivary gland (SG), and lung to look for organ specific markers of inflammation. Here, we give an overview of research involving SLE and organ specific research on biomarkers, transcriptomics/scRNAseq, and molecular imaging.

## Inclusion and exclusion criteria

While SLE is defined as an autoantibody and immune complex disease, most of its organ manifestations are inflammatory. That is why we in this review have focused on the inflammatory milieu in the different organs included in our investigation. Thus, we used inflammation and human as a prerequisite in most of our PubMed searches. We included research on other autoimmune diseases to compare the findings in SLE, especially where there is a lack of research on SLE. To limit the results and focus on human research we excluded paper containing virus, animal, and cancer research (Supplementary Table S1). We have also focused on the articles published the last 5–10 years.

## Inflammation in different organs of autoimmune and rheumatic diseases

**Figure 1:**
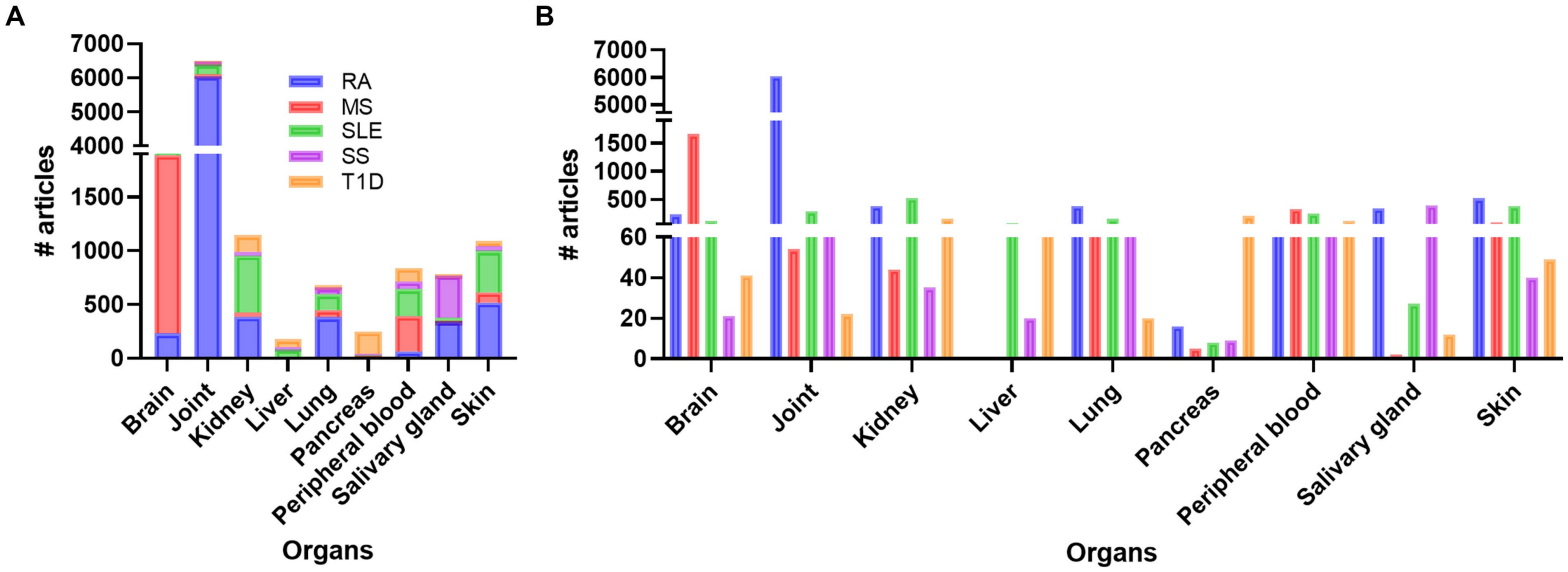
Overview of the number of publications on disease, inflammation, and organ involvement **(A)**. In **(B)** the individual results of each disease related to organs and number of publications are shown. RA, rheumatoid arthritis; MS, multiple sclerosis; SLE, systemic lupus erythematosus; SS, Sjogren’s syndrome; T1D: type 1 diabetes.

Doing a PubMed search on inflammation, different diseases, and organ, revealed that a vast amount of research has been performed on joint compared to other organs like brain, skin, kidney, SG, lung, and pancreas, respectively (Figure 1; Supplementary Table S1). Organ affection in SLE include all the above-mentioned organs, in addition to circulating immune cells in peripheral blood. Other autoimmune and rheumatic diseases are more organ specific, like multiple sclerosis (MS) and brain, and Type 1 Diabetes (T1D) and pancreas, while others like Rheumatic arthritis (RA) and Sjogren’s syndrome (SS) can be both organ specific and systemic. The search confirmed the typical organ manifestation of the different diseases as most of the papers in rheumatoid arthritis (RA) were found in joint, in MS the brain had more publications, SG in Sjogren’s syndrome (SS), pancreas in Type 1 diabetes (T1D), and the kidney in SLE patient (Figure 1). The results revealed that, despite being a systemic disease affecting multiple organs, most of the published articles on SLE involved kidney, skin, and joint, and fewer papers on the other organs, especially SG, and pancreas (Supplementary Table S1; Figure 1).

## Disease and organ specific biomarkers and advanced methods

**Figure 2:**
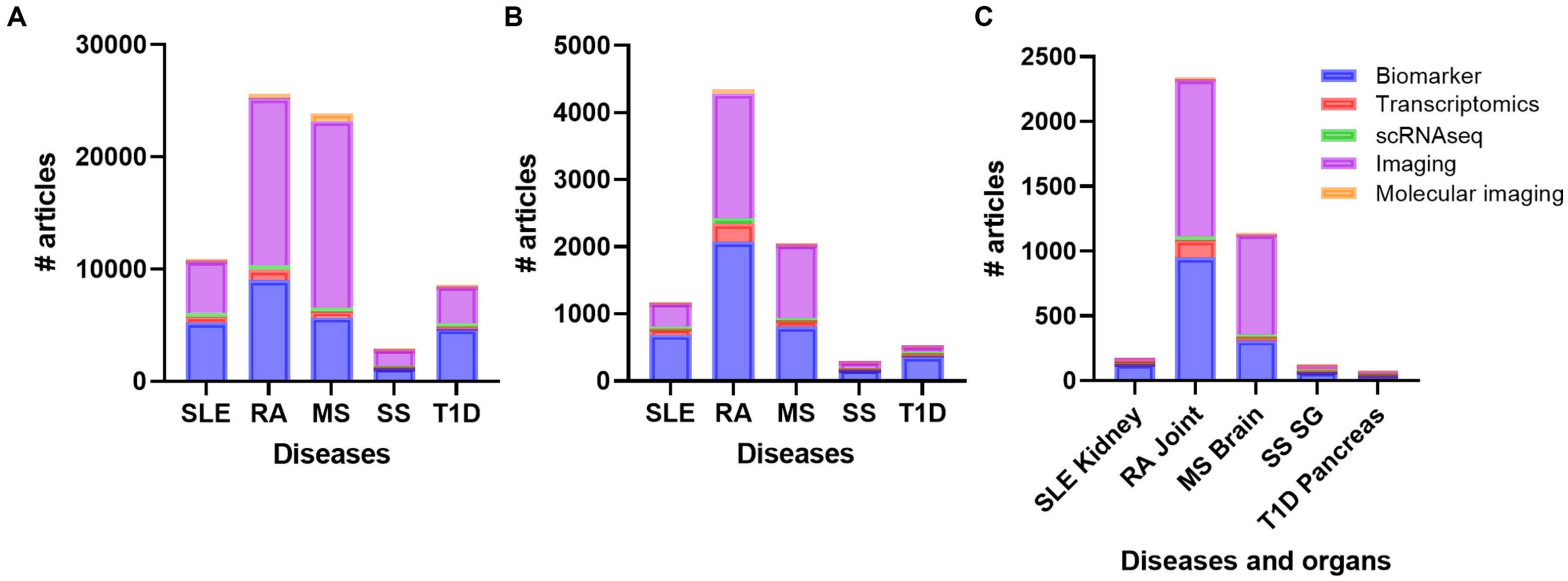
Overview of the number of publications on diseases **(A)** diseases and inflammation **(B)** and diseases, inflammation and organs **(C)** with the topics biomarker, transcriptomics, single cell sequencing, imaging, and molecular imaging. RA, rheumatoid arthritis; MS, multiple sclerosis; SLE, systemic lupus erythematosus; SS, Sjogren´s syndrome; T1D: type 1 diabetes; SG, salivary gland.

To get an overview of different organ specific biomarkers in the field of autoimmune and rheumatic diseases, we did several defined PubMed searches on the diseases SLE, RA, MS, SS, and T1D. These searches revealed that there are more studies performed on MS and RA compared to SLE, SS, and T1D on every search we did, respectively (Figure 2A). We did searches on advanced methods such as transcriptomics, single-cell sequencing, imaging, molecular imaging, and biomarkers (Supplementary Table S2; Figure 2A). When we included inflammation, the number of papers decreased, but the overall results showed the same trend (Supplementary Table S3; Figure 2B). These results were also supported when we added the different organs to the search terms (Supplementary Table S4; Figure 2C). Biomarker and imaging got the most hits, and after including specific organs, the findings even increased the disparities in number of works done between RA and MS compared to the other diseases (Figure 2C).

## Biomarkers in SLE

Autoantibodies have been widely used as biomarkers for rheumatic [reviewed in (11)] and autoimmune diseases [reviewed in (12)] such as SLE [reviewed in (13)]. Anti-nuclear antibody (ANA), anti-dsDNA, anti-Sm, antiphospholipid, and low C3 and/or low C4 are used in clinical practice today as diagnostic biomarkers of SLE [reviewed in (14)]. Although there are several publications available on novel biomarkers for use in diagnosis [(15) and references therein], and monitoring [reviewed in (14, 16)] of SLE, few of them are in use in clinical practice. The heterogeneity of SLE makes it difficult to diagnose and monitor the disease progression. Performing a PubMed search on inflammation, SLE and biomarker, gave 689 results, of which 488 articles were published the last ten years (Supplementary Table S3; Figure 2B). Of these, 91 publications were reviews, compared to 121 in total.

Since a primary goal for developing new biomarkers is to identify non-invasive markers most of the works utilized blood or urine from patients with SLE. Searching PubMed for human SLE, peripheral blood and biomarker, excluding virus and cancer, gave 71 results, including five review papers, while the same search with urine instead of blood revealed 66 results, including eight review papers. Several studies have taken advantage of proteomics in the identification of new biomarkers. Adding proteomics to our PubMed searches revealed 31 original articles and 8 reviews, and only 6 articles when inflammation was included. The last five years revealed few experimentally work on serological and urinary biomarkers for the detection of inflammatory human SLE (Table 1), nearly all of them detecting active LN. Most of the identified biomarkers included in this table are part of molecular pathways involved in the pathogenesis of SLE. However, some of the markers such as albumin, ceruloplasmin, transferrin, lipocalin-type prostaglandin D2 synthase (LPGDS), and different collagen types are most likely a response to or caused by the inflammatory process. A recent review by Fasano et al. covers different tissue, serological, urinary, and cellular biomarkers related to molecular pathways in SLE (48). Since late 1980s, work has been published on double-negative T cells. These cells are implicated in inflammation, immune disorders, cancer, and kidney as reviewed in (49, 50). These cells have been observed in SLE, RA, and SS where they are described to have a worsening role, and in T1D where they are described to be protective [(51) and references therein]. In SLE, double-negative T cells have been suggested as potential serum biomarkers for SLE patients, possibly with kidney involvement (52). Exosomes are spherical lipid bilayer vesicles consisting of lipids, proteins, nucleic acids, and other bioactive compounds. Exosomes have been implied to be involved in immune responses and regulating the development of autoimmune disease through the transfer of signaling molecules as reviewed in (53). Studies have investigated exosomes and its components, both in serum and urine, and suggested them as potential biomarkers in SLE (54, 55), reviewed in (56, 57).

**Table 1:** Summary of the proposed serological and urinary markers in inflammatory, human SLE revealed in this review.

Biomarker	Detects	References
Serological		
VCAM1	Predict nephritic flare, active non-renal lupus, and CKD	(17)
ICAM-1	Nephritic flare, LN remission	(17)
Syndecan-1	Predict nephritic flare, active LN, active non-renal lupus and patients with non-lupus CKD, renal interstitial inflammation	(18)
Hyaluronan, Thrombomodulin	Active LN,LN patients in remission and non-lupus CKD, renal chronicity	(18)
PRO-C6, Collagen Type III and IV	Kidney fibrosis	(19)
IL-35	Renal involvement	(20)
MALT1	Severity and inflammation in LN	(21)
TNF-α, p-albumin	Differentiate between SLE patients and controls, and indicators of disease activity	(22)
IP-10, IL-1α, IL-6, TNF-α, and ESR	Joint involvement/active arthritis	(22)
IL-16, anti-dsDNA and IL-10	Active LN	(22)
cGAS, IFI16	Correlate with SLE disease activity	(23)
L-alpha-aminobutyric acid, dehydroascorbic acid, glycine, and L-tyrosine	Skin manifestation	(24)
*NAMPT,* eNAMPT	Increased alveolar hemorrhage and lung inflammation	(25)
IL-26	Active disease	(26)
S100A8/A9 + S100A12	Response to treatment of rituximab	(27)
CD5L	Monitoring of SLE and therapeutic efficacy	(28)
*TNFSF13B* (BAFF) and *OAS1*	Diagnostic marker SLE and metabolic syndrome	(29)
EGF, Lipocalin-2/NGAL, uPA, IL-18	Biomarkers of renal pathology	(30)
IgA2 anti-dsDNA ab	Active renal disease	(31)
Urinary		
CD163	Active LN, higher CKD stage	(32, 33)
IL-16	Proliferative LN	(33, 34)
MCP-1 (CCL2)	Active LN, non-response, LN flare, loss of kidney function	(35, 36)
EGF	Biomarker of CKD progression in patients with glomerular disease (in adults and children)	(37, 38)
Serpin-A3	Active LN, proliferative LN, response to therapy in proliferative LN	(39)
Leukocytes	Active LN, response to treatment	(40)
Ig binding protein 1	Active LN	(41)
C3M	Kidney fibrosis	(19)
TWEAK	Active LN	(42)
Semaphorin3A	Renal involvement in SLE	(43)
suPAR	LN activity	(44)
S100	Active LN	(27)
Transferrin, AGP-1, MCP-1, sVCAM-1	Higher in SLE patients	(45)
Transferrin, LPGDS, ceruloplasmin, MCP-1 + sVCAM-1	Active LN	(45)
LPGDS, transferrin, AGP-1, ceruloplasmin, MCP-1 + sVCAM-1	Response to rituximab treatment	(45)
Angiostatin, CXCL4, VCAM-1	Biomarkers of LN	(46)
Exosomal miR-146a	LN and SLE flares	(47)

Results from the last 5 years. LN: lupus nephritis; CKD: chronic kidney disease; SLE: systemic lupus erythematosus.

Plasma cytokines are also potential biomarkers in patients with SLE. Multiplex studies performed by Idborg et al. (22), showed that TNF-α and p-albumin worked as potential biomarkers to differentiate between SLE patients and controls, as well as indicators of disease activity. In addition, they showed that for SLE patients with joint involvement/active arthritis, IP-10, IL-1α, IL-6, TNF-α, and ESR were significantly increased compared with patients without these manifestations. They also investigated whether organ specific, active disease was associated to selected cytokines. P-albumin and TNF-α showed the highest association to kidney involvement, while IL-16, anti-dsDNA and IL-10 were elevated in active nephritis. Previously, Pentraxin 3 (PTX3) has been suggested as a biomarker of tubulointerstitial damage. Indeed, in a large, multicenter cross-sectional study, ELISA showed that higher PTX3 levels were found both in serum and urine of active phase LN patients (58). Peripheral blood mononuclear cells (PBMC) are also an easily accessible source of potential biomarkers. In a recent study by Fu et al. cyclic GMP-AMP synthase (cGAS) and interferon-I-inducible protein 16 (IFI16) were found to correlate with SLE disease activity (23). An interferon (IFN) signature with an increased expression of type I IFN-regulated genes, are well-known in patients with SLE. Jiang and colleagues (59) utilized bioinformatics and machine learning to identify biomarkers for use in SLE diagnosis. They identified ten potential diagnostic biomarkers and found one, IFI44, a type I IFN signature gene, that had the best potential of being an optimal diagnostic biomarker for SLE. However, the process of validating these findings experimentally and clinically remains to be done.

## SLE organ specific biomarkers

**Figure 3:**
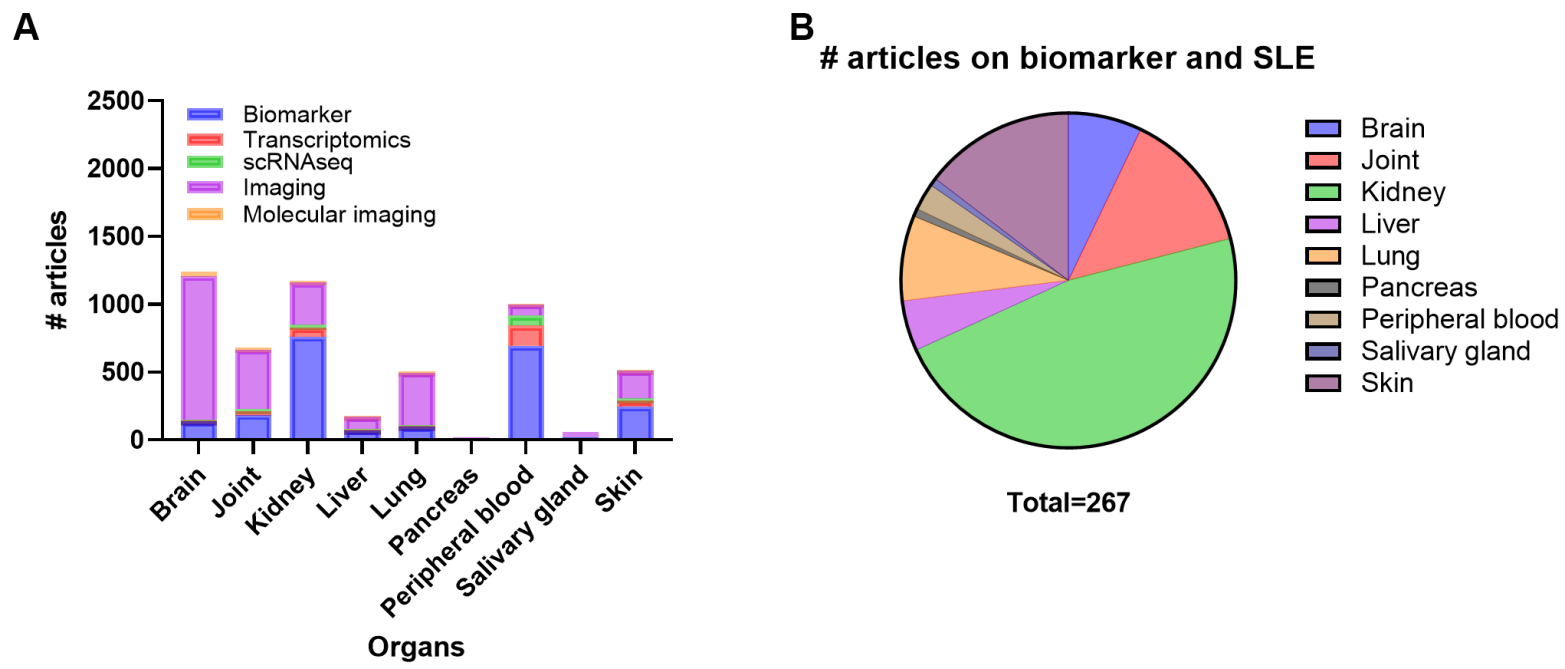
Overview of the number of publications on systemic lupus erythematosus (SLE), organ involvement and biomarker, transcriptomics, scRNAseq, imaging, and molecular imaging **(A)** and number of publications on biomarker in SLE with inflammation and organ involvement **(B)**. scRNAseq, single cell RNA sequencing.

Monitoring disease activity and organ damage in SLE is challenging due to the lack of dependable biomarkers and disease heterogeneity. Both permanent organ damage and ongoing systemic and organ-specific inflammation can be hard to differentiate. Thus, organ-specific biomarkers would be precious for systemic diseases such as SLE. A search on SLE and organs and biomarkers demonstrated that most of the research has been performed on kidney, and peripheral blood, followed by skin, joint, brain, lung, and liver, while very few articles on biomarkers in SGs and and pancreas exist (Supplementary Table S5; Figure 3A). Here we searched PubMed for articles comprising SLE, inflammation and different organs such as kidney, skin, joint, lung, brain, liver, pancreas, and SG looking for SLE biomarkers specific for the different organs (Figure 3B). From this search, we did not find any experimental papers on joint, liver, and pancreas.

## SLE biomarkers in skin, lungs, brain, and salivary glands

Xie et al. identified L-alpha-aminobutyric acid, dehydroascorbic acid, glycine, and L-tyrosine as serum metabolites with diagnostic potential for SLE patients with skin lesions (24). Cutaneous lupus erythematosus has been found to be associated with an increase of invariant natural killer T (iNKT) cells expressing CCR4 at the site of active dermal cutaneous lesions, with a subsequent deficiency in circulating iNKT cells (60). One study also showed serum DNase I activity and apoptotic index evaluated by immunohistochemistry to be possible biomarkers of cutaneous lupus erythematosus (61). Nitric oxide (NO) in exhaled air has been shown to be significantly increased and correlated with disease activity and might be used as a marker for lung involvement in patients with SLE (62). In one study from Tumurkhuu et al. (25), SLE subjects showed highly significant increases in blood *NAMPT* mRNA expression and eNAMPT protein levels compared to healthy controls. In mice, this is shown to be associated with increased alveolar hemorrhage and lung inflammation. Going through publications on brain and SLE, we excluded publications on SLE and pregnancy. However, one publication on childhood-onset systemic lupus erythematosus (cSLE) showed that serum levels of S100A8/9, S100B, NGAL, aNR2-AB and aP-AB and combinations of those, could detect neurocognitive deficits in cSLE (sensitivity: 100%; specificity 76%) in exploratory analysis (63). Another work showed that Progranulin (PGRN) was moderately increased in cerebrospinal fluid (CSF) of SLE patients (64). Many SLE patients are diagnosed with secondary Sjogren’s syndrome. In one study, two of 34 patients were diagnosed with SLE and secondary SS. The authors showed that with higher pathologic grade, the presence of Sjogren’s-syndrome-related antigen A (SSA) or a higher titer of ANA were significantly associated with the overexpression of TRAIL, MMP-3, or ICAM-1 in the SG mononuclear cells in patients with SS (65).

## Kidney specific biomarkers in SLE

Kidney is definitively the organ that has been most extensively researched in SLE. LN is also one of the most serious manifestations of SLE. A retrospective study by Mao et al. where they did proteomics on kidney biopsies, revealed renal mTORC1 activation as a possible biomarker for disease activity and prediction of clinical prognosis in LN patients (66). Another study showed urinary C3M, and serum derived PRO-C6, Collagen Type III and VI, to correlate with kidney fibrosis in SLE patients (19). Studies on serum Interleukin-35 (IL-35), and mucosa-associated lymphoid tissue lymphoma translocation protein 1 (MALT1) propose low IL-35, and high MALT1, respectively, to be potential biomarkers for renal involvement in SLE patients (20, 21). Biomarkers that can be indicative of response to treatment are useful. Indeed, Shipa et al. did a study where they identified serum IgA2 anti-dsDNA antibody concentrations that could predict a clinical response to belimumab after rituximab treatment (31). The presence of anti-ribosomal P antibody (anti-P) was in a study with 79 patients shown to be associated with better histological findings. In addition, at a median follow-up time of 47 months, anti-P-positive patients shown better renal outcomes than those without anti-P (58). A study by Sun et al. showed that urine tumor necrosis factor-related weak inducer of apoptosis (TWEAK) may be utilized as a biomarker of LN activity (42). The same was shown in a study on urinary DNase I, showing that its expression and activity declined with disease progression (67). A marker of podocyte injury was investigated by Bouachi et al. (68). Here they found that CMIP induction in LN appears constrained to non-proliferative glomerulopathies and may define a specific pattern of injury to these cells (68).

Due to the heterogeneity of SLE, there exist few biomarkers with the potential to capture the complexity of disease monitoring. Therefore, we need a combination of biomarkers and/or organ-specific biomarkers to follow disease progression. This review reveals that kidney, skin, and joint, respectively, have traditionally received the most attention in SLE because they are the most affected organs with frequent complications, but little progress has been achieved on organ-specific diagnostic or therapeutic markers.

## SLE transcriptomics

**Figure 4:**
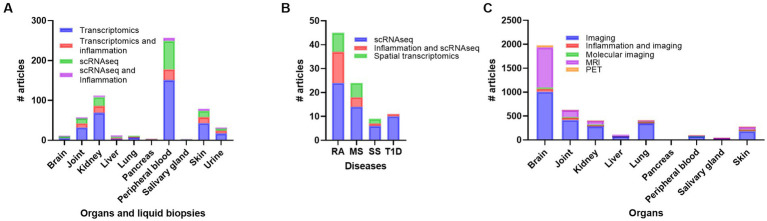
Overview of number of publications on transcriptomics, transcriptomics + inflammation, scRNAseq, and scRNAseq + inflammation in systemic lupus erythematosus (SLE) **(A)**, number of publications on scRNAseq, scRNAseq + inflammation, and spatial transcriptomics in rheumatoid arthritis (RA), multiple sclerosis (MS), Sjogren’s syndrome (SS) and Type 1 diabetes (T1D) **(B)**, and number of publications on imaging, imaging + inflammation, molecular imaging, magnetic resonance imaging (MRI) and positron emmission tomography (PET) in SLE **(C)**. scRNAseq, single cell RNA sequencing.

Transcriptomic data analyses offer a unique insight into processes involved in different autoimmune disease. Since 2005, transcriptional analyses on human SLE and inflammation have been published in 85 research papers and 10 review papers (Supplementary Table S3). As for biomarkers, most of the transcriptomic research has been performed on peripheral blood followed by kidney, skin and joint (Supplementary Table S6; Figure 4A). PBMC analyses have revealed increased differences in the composition of immune cells (B, T, and myeloid cells), type 1 IFN signature, chemokines, and different transcriptional levels of cytosolic RNA and DNA sensors (69–76). Especially, it seems to be a correlation between reduced circular levels of naïve T cells and increased T cells in the different organs affected. A dysregulation of T regulatory cells vs. T helper cells (Th1, Th2 and Th17) is known to be important for the pathogenesis of SLE, and the involvement of Th1, Th2 and Th17 in SLE has recently been thoroughly reviewed (77). Transcriptomic analyses of circulating immune cells found upregulation of Th17 related genes *CXCL1, ICAM1, IL10, IL5, IL8, ISG20, JAK2*, and down regulation of *CD28, CD40LG, S1PR1, IL17RE, IL23R, RORC* genes (78).

The IFN signature in SLE can divide the patients into two groups: SLE1 with low levels IFN induced gene expression and SLE2 with high levels of IFN induced gene expression (79). In SLE2 patients a subset of cytotoxic CD4^+^ T cells has been identified (72). Buang et al. found a downregulation of mitochondria-derived genes and metabolic pathways T cells of IFN high SLE patients (80). A transcriptome-wide association study (TWAS) was performed using a model on blood B cells, T cells, monocytes, NK cells and PBMC. Here they identified *BANK1, IRF5, BLK, NCOA2, WDFY4, SLC15A4 and RASGRP1* as differential expressed genes and potential biomarkers (81).

In the kidney, the early articles on transcriptomic data identified specific cellular expression of glomerular cells and selected inflammatory markers of infiltrating cells using qPCR analyses (82, 83). The newly published research includes transcriptional profiling of kidney tubular cells and immune cell infiltration in patients with LN (84–89). The main findings identify, like in peripheral blood samples, inflammatory markers such as type I IFNs, IL-18, TNF, immune cells including myeloid cells, T cells, natural killer cells and B cells, plasma cells and chemokines and chemokine receptors. Especially chemokine receptors *CXCR4* and *CX3CR1* expression by kidney immune cells seems to be candidates for use as biomarkers (85). In the skin of SLE and cutaneous lupus erythematosus (CLE) a hypersensitive response to IFNs was observed in keratinocytes (90).

Biopsies are only taken from patients with suspected kidney disease, but many SLE patients may have changes within the kidney long before they develop clinical detectable inflammation. Several studies have analyzed the transcriptomic profile of cells and cellular casts in urine from patients with SLE and LN (33, 83, 85, 91). Urinary transcriptomics reviled both a podocyte cell specific signature and an enrichment of immune cell types reflecting the inflammation of the kidney (83). Arazi et al. compared immune cells from urine samples with the kidney samples and found that the main fraction of urine cell was phagocytic CD16+ macrophages followed by M2-line CD16^+^ macrophages, CD56^dim^ CD16^+^ NK cells, inflammatory CD16+ macrophages, tissue resident macrophages, and conventional DCs (85). A study analyzing both proteomics on urine samples and transcriptomics on kidney biopsies found increased urine levels of IL-16, CD163 and TGFB1 and an increased expression of *IL16* in most of the infiltrating immune cells, *CD163* in a subset of myeloid cells, and *TGFB1* was mostly expressed by NK cells (33). In a recent article addressing the proteomics in paired urine and biopsy from SLE and LN patients, they identified over 112 urine analytes and especially found proteins involved in granulocyte-associated and macrophage-associated pathways, including CD163 (92).

## SLE single cell sequencing (scRNAseq)

ScRNAseq has mostly been performed on peripheral blood, with fewer studies on kidney, skin, and joint samples from SLE patients (Figure 4A). ScRNAseq performed on kidney (84, 85), skin (84, 93), and PBMC (94) revealed common factors including different immune cell types and type I IFN signature. The chemokines and chemokine receptors expression were linked to tubular cells (kidney) or keratinocytes (skin) and immune cells (84). Guo et al. identified a reduction of CXCR5^+^ T cells in SLE patients and found an exhausted regulatory CD4^+^ T cell subset in PBMC from SLE patients induced by type 1 IFN signaling (95).

In the skin, the same types of T cells including CD4^+^ and CD8^+^ memory T cells, Tregs, cytotoxic T cells, Th cells, and others were found to express increased levels of IFN-genes, but appeared less activated and cytotoxic and did not show an exhausted phenotype as T/NK cells from the kidney of LN patients (87). Zheng et al. analyzed cells from different cutaneous lesions of SLE patients and found both similar and differences in cell compositions of skin biopsies from patient with discoid lupus erythematosus (DLE) and SLE (96). Similarities included the proportion of macrophages and dendritic cells in both epidermis and dermis, while differences in T, NK and B cells were prominent in dermis as DLE patients had more T, NK, and B cells. DLE skin biopsies showed a more organized accumulation of immune cells (96).

Li et al. (97) performed both proteomics and scRNAseq on PBMC from SLE patients. They identified, by using machine learning, combinations of biomarkers that could diagnose SLE and the cellular source of these proteins was determined using scRNAseq (97). The six-protein combination included IFN inducible genes (IFIT3, MX1, TOMM40, STAT1, STAT2, and OAS3) and the nine- protein combinations included mitochondrial enzymes, adhesion molecule, kinases and other proteins (PHACTR2, GOT2, L-selectin, CMC4, MAP2K1, CMPK2, ECPAS, SRA1, and STAT2) (97). Genes in the six-protein combinations were shown to be mostly upregulated in memory B cells, while the nine-protein combination genes were upregulated in CD14 monocyte clusters. Tang et al. performed scRNAseq on PBMC from SLE patients to analyze the expression of acetyl transferases that can regulate cyclic GMP-AMP synthase (cGAS) a cytoplasmic DNA sensor (75). They identified an upregulation of histone acetyltransferase *KAT2A* in SLE patients and showed that the pathway was increased in specific subtypes of myeloid dendritic cells (DC), monocytes, T and B cells observed in SLE patients with a high SLEDAI score (75).

Itotagawa et al. used existing scRNAseq data to identify the BAFF producing cells in the kidney of SLE patients and linking the urinary BAFF levels to LN (98). The usage of available scRNAseq data together with advanced proteomics and the development of AI pipelines offers a deeper understanding of the complexity of disease progression. Wang et al. used a combination of machine learning algorithm and scRNAseq analysis to find possible biomarkers and identified both protein and gene expression of TNFSF13B and OAS1 (29). As mentioned under the section on biomarkers, a study using scRNAseq data from SLE patients (99) identified IFI44 as possible biomarker for SLE (59). However, linking these findings to the existing clinical parameters and developing new and better disease biomarkers is a challenge.

## Transcriptomics and scRNAseq in other autoimmune diseases

Transcriptomics and scRNAseq data from other autoimmune diseases may fill in the lack of data in SLE. A PubMed search on RA, MS, SS, T1D and scRNAseq and scRNAseq + inflammation, including only original articles with new scRNAseq data gave 24 and 13 results on RA, 14 and 4 results on MS, 6 and 1 results on SS and 10 and 1 results on T1D, respectively (Figure 4B). In RA, CD4^+^ T cell clones in peripheral blood and synovial tissue were shown to be under constant activation and had a senescent phenotype (100). Some studies have analyzed and compared the gene expression profiles between autoimmune diseases. Tuller et al. analyzed gene expression profiles of PBMC from MS, SLE, juvenile (J)RA, crohn’s disease (CD), ulcerative colitis (UC) and T1D patients compared to healthy controls (101). A common trend was observed for the chemokines *CXCL1-3, 5, 6,* and *IL8* in addition to differentially expressed genes involved in cell proliferation, inflammatory response, general signaling cascades, and apoptosis. The authors suggest that despite the similarities, the results indicate an activation through different sub-signaling pathways (101). In another study, distinct DC clusters characterized by up-regulation of *TAP1*, *IRF7*, and *IFNAR1*, were identified in systemic autoimmune diseases and *PTPN6*, *TGFB*, and *TYROBP* were shown to be downregulated in DCs in T1D (73). Liao et al. used existing scRNA data on fibroblasts from RA and osteoarthritis from synovial tissue (102). They identified CCL2 and MMP13 as possible diagnostic and therapeutic markers. Another study focusing on transcription factors revealed five regulators BATF, POU2AF1, STAT1, LEF1 and IRF4 specific for RA fibroblast-like synoviocytes (103).

Stephenson et al. used a low-cost microfluidic instrumentation to perform scRNAseq analyses and identified synovial fibroblast (SF) subtypes in addition to well-known immune cell clusters (104). The fibroblast subtype (*THY1(CD90)*^+^*HLA-DRA^hi^*) was verified and linked to *L1B*^+^ pro-inflammatory monocytes, *ITGAX*^+^*TBX21*^+^ autoimmune-associated B cells and *PDCD1*^+^ T peripheral helper (Tph) and T follicular helper (Tfh) (105). In synovial macrophages isolated from RA patients, the expression of signaling lymphocytic activation molecule F7 (SLAMF7) was identified as a marker for super activated macrophages (106), and upregulation of CD86 and CD206 was observed in cells from the synovium lining layer (107). Yamada et al. analyzed synovial scRNAseq data identifying a subset of pre-dendritic cells possibly predicting resistant to treatment (108). Another study analyzing the CD4^+^ T cell subsets in RA identified two Tph states (*CXCL13* high and low) and a cytotoxic CD4^+^ T cell subset where all expressed GPR56 (*ADGRG1*) (109). Moon et al. used scRNAseq and TCR sequencing on CD8^+^ T cells from blood and found seven distinct clusters of CD8^+^ T cells (Naïve, Memory, *TCRgd*^+^, *GZMK*^+^*, GZMB*^+^ *GNLY*^+^*, GZMB*^+^ *KIR*^+^ *and CCR6*^+^ *CD161*^+^) where cluster *GZMB*^+^ *KIR*^+^, *TCRgd*^+^, and memory were increased, and cluster *CCR6*^+^ *CD161*^+^ were decreased in RA patients compared to healthy controls (110). Two groups have studied different classes of monocytes and macrophages isolated from blood of RA patients to analyze response to treatment and the function of macrophages, respectively (111, 112). Several studies have used existing and/or own scRNAseq data to identify RA mechanisms or disease markers. An article by Orange et al. showed increased expansion of preinflammatory mesenchymal cells (113), while Micheroli et al. identified four distinct SF clusters (114). Yang et al. studied scRNAseq datasets for SFs revealing *Fibronectin-1* as an important gene in relation to RA disease progression (115). A recent review discusses the development of residential memory T cells in synovial tissue in RA patients and indicate a role for these cells in the transition from acute to chronic inflammation (116).

ScRNAseq on cells from CSF and blood in MS patients demonstrate a strong immune cell profile like the signatures seen in other autoimmune diseases. Especially B cells are increased in CSF in addition to CD4^+^ and CD8^+^ T cells (117). Several studies have also analyzed microglial cells using scRNAseq [reviewed in (118)]. In SS scRNAseq analyses on PBMC, monocytes were the abundant cell type and the expression of transcription factor *CEBPD* and tumor necrosis factor (ligand) superfamily, member 10 *TNFSF10* (TRAIL) in CD14^+^ monocytes were increased (119, 120). Hou et al. showed an increased percentage of Tregs while CD8^+^ T cells, mucosal associated invariant T cells (MAIT) and double positive CD4^+^ CD8^+^ T cells were reduced in SS compared to healthy donors (121). However, a recent publication from Xu et al. showed increased expression of CD8^+^ T cells in patients with SS (122). These granzyme K^+^ (GZMK^+^) CXCR6^+^ CD8^+^ T cells from blood were similar to a distinct group of tissue-resident memory T cells. The same study showed an increased expression of circulatory IL-15, possibly promoting this specific differentiation of the CD8^+^ T cells (122). Hong et al. identified an increased cytotoxic CD4^+^ T cell population in addition to increased *IL1b* in macrophages, *TCL1A* in B cells, and like in SLE an increased expression of IFN responsive genes in most of the immune cells analyzed (123).

A recent review paper gives an overview of the existing sequencing platforms and especially on how scRNAseq has been used in over 41 autoimmune diseases (7). The results from scRNAseq identifies both common and disease specific subtypes of cells triggered by common and different signaling pathways. Here they conclude that the identification of specific disease-causing cells using scRNAseq in combination with other methods is a prerequisite for developing more effective targeting treatments with less side effects (7). Ma et al. used scRNAseq data from SLE patients (124), and compared it to RNA bulk sequencing on PBMC from SLE, RA and MS patients and healthy donors by using machine learning to develop a method to identify characteristic of SLE and to distinguish SLE patients from healthy donors (125). Their mathematical model could also identify patients with RA and MS and proves that the usage of existing scRNAseq and the development of machine learning models could be used to develop efficient tools for diagnosis of chronic autoimmune diseases.

## Spatial transcriptomics

Spatial transcriptomics is a new technique applying scRNAseq on tissue samples allowing visualization and quantitative analyses of spatial gene expression patterns in individual tissue sections at near single-cell resolution (126). We found 8 papers on spatial transcriptomics and RA, 6 on MS and 2 on SS (Figure 4B). However, many of these were review papers. Most of the research involving spatial transcriptomics has been published on synovial tissue from RA patients (127–129) and brain tissue from MS patients (130, 131). So far, no spatial transcriptomics analyses have been published on tissue from SLE or LN patients. However, research have been published on normal kidneys (132), transplanted kidneys (133), and some review papers have given an overview of the use of spatial transcriptomics and analyzing kidney diseases (134–138). Spatial transcriptomics on normal tissue may offer a new understanding of tissue cells and residential immune cells. Madissoon et al. used multi-omics single cell/nuclei and spatial transcriptomics to define the tissue architecture of lungs and airways (139). The results reviled a new specific niche for immune cells in the lungs and may be used further in analyzing molecular and cellular changes during different lung diseases.

Several review papers have addressed the advantage of spatial transcriptomics in different autoimmune diseases (116, 136, 138, 140). The development of disease pathology Atlases based on bulk RNAseq, scRNAseq, and spatial transcriptomics, machine learning models, and the use of AI to combine clinical and multiomics data, will be crucial in the future research on chronic autoimmune diseases including SLE. Especially, to identify clinically relevant patterns in the abundance of information available.

## SLE and organ imaging

Imaging, inflammation and SLE reviled 357 papers and 26 of them included kidney (Supplementary Table S7). Papers involving imaging and brain (81), joint (70) and skin (62) were prominent with lung (57) at the same level as kidney (Figure 4C). The imaging methods used included mostly histological or immunohistology assessment of biopsies. Gallium-67 scintigraphy was previously performed on LN patients before and after biopsy to predict response to therapy (141). Recently, a new study analyzed over 250 biopsies from LN patients that underwent renal gallium scans before or after biopsy (142). They found an association of renal gallium uptake and active inflammation measured by hypercellularity, neutrophil infiltration and changes in the activity index.

Research on SLE and molecular imaging consist of 20 articles where 3 are review papers. Including inflammation in the search reduced the papers to 4 (2 reviews). The reviews cover the use of non-invasive imaging of LN and neuropsychiatric SLE and especially the use of PET (143, 144). A more specific search on inflammation, SLE and MRI found 139 papers involving MRI on brain (74), joint (52), skin (10), kidney (6), SG (1) (Supplementary Table S8; Figure 4C). Most of the MRI has been conducted on brain and the association of inflammation and functional and structural brain changes in neuropsychiatric SLE (143, 145). A broader search on SLE, MRI and kidney reviled several research papers on the use of multiparametric MRI including blood oxygen level-dependent (BOLD) imaging by T2* mapping, magnetic resonance elastography (MRE) by tomoelastography, diffusion tensor imaging (DTI) and diffusion-weighted imaging (DWI) for detecting nephropathy in LN patients (145–151). Here BOLD, DTI, and DWI imaging may determine the disease severity, effect of treatment, and outcome of the disease (151).

PET/CT and PET/MRI has been used to image brain (144, 152), kidney (153), and lung (152) of SLE patients [reviewed in (143, 154)]. Most of the research used ^18^FFDG, but in brain 2 studies used TSPO targeted PET/MRI to image hippocampal neuroinflammation and were able to detect signal alterations [reviewed in (155)]. However, Nwaubani et al. reviled a lack of research to examine hippocampal subfield separatley, and suggests new methods with higher resolution acquisition or post-processing teckniques (155). Despite some preclinical studies using PET/CT or PET/MRI in kidneys, none has been performed on SLE or LN patients. However, Carlucci et al. performed PET/CT with 18F-FDG to quantify vascular inflammation and significant increased aortic inflammation in SLE patients compared to healthy controls (156).

The lung involvement in SLE has recently been reviewed by Shin et al. revealing several imaging methods (157). Chest X-ray (CXR), CT, technetium-99 m hexamethylprophylene amine oxime perfusion scan, and ^18^FFDG PET are used to diagnose pleuropulmonary involvement in SLE. The involvement includes lupus pleuritis, pleural effusion, acute lupus pneumonitis, shrinking lung syndrome, interstitial lung disease, diffuse alveolar hemorrhage (DAH), pulmonary arterial hypertension, and pulmonary embolism (157).

This review reviled many imaging methods that have been performed on SLE patients with brain and lung involvement. However, despite being a systemic disease, only few imaging methods tested have focused on the detection of systemic inflammation in SLE. One of the reasons is the lack of specific markers for systemic inflammation. Another issue is the filtration of tracers by the kidney, making imaging of this organ difficult. The development of new specific long-lived isotopes (89Zr, 68Cu) (158, 159) conjugated to different immunobiologicals (ligands, antibodies, Fabs) may generate tracers that can detect changes within the kidney and detect early inflammatory events (160). *In vivo* imaging using PET/MRI with specific tracers to detect tissue damage or immune cell involvement may be used as a diagnostic tool to detect inflammatory kidney diseases at an early stage, as a non-invasive method to follow disease progression, and to more specific, tailored/personalized treatment. A tracer that detects systemic inflammation will be beneficial for other autoimmune diseases.

## Conclusion

Publications comprising advanced methods and biomarkers that can be used for various diseases, but with a focus on SLE, have been reviewed. Is it possible to find common methods and biomarkers across different diseases, and is it possible to find organ specific biomarkers to monitor organ inflammation? A similar approach has been used in the Taxonomy, Treatment, Targets and Remission (3TR) study where they aim to investigate the mechanisms of response and non-response to treatment in chronic autoimmune diseases (MS, SLE and RA), inflammatory bowel diseases (Ulcerative Colitis and Crohn’s Disease), and respiratory diseases (Asthma and Chronic Obstructive Pulmonary Disease).^1^ Since SLE is a systemic disease, it could fit as a model disease to use in such studies. A recent review from the 3TR study on LN gives an overview of biomarkers that is suitable for diagnostics, determine disease activity and organ damage, determine the response to therapy, and biomarkers to be used to determine the prognosis (161).

As heterogenous as SLE is, one ideal biomarker or method does probably not exist for disease diagnosis and monitoring. Instead, we ought to look for biomarkers and methods that have specificity and sensitivity for distinct organ involvement. We suggest that utilizing results from other autoimmune diseases with different organ manifestations would be of interest in SLE. Probably organ-specific biomarkers can be exploited across different diseases. Indeed, Johnson et al. showed that there are selected biomarkers that are more valid for diabetic kidney disease compared to LN, but none of them could distinguish between these two diseases (30). Zhang and Lee used a combined strategy utilizing multi-omics data analysis and computational methods and identified specific variable (V) and joining (J) genes in both T cell receptor and B cell receptor in SLE and RA groups (162). In addition, looking for a combination of biomarkers, composite biomarkers, and combined strategies with biomarkers, new methods and clinical measurements is perhaps the best strategy ahead. Using transcriptomics combined with principal component analyses (PCA)/AI to search for inflammatory markers in blood and urine can substitute for invasive kidney biopsy and allows physicians to monitor treatment and disease progression. Liquid biomarkers enable non-invasive, real-time detection of circulating markers in blood or urine. By its nature, urinary biomarkers are by far the best alternative when it comes to noninvasive biomarkers. Especially for the monitoring of kidney affection, urine is probably the best source for easy follow-ups. However, multiomics studies generate a lot of data, but there are still few, specific studies on autoimmune diseases. Also, there is a need to combine, compare, and extract the results before verifying their importance in the clinic. These issues must be addressed before we can develop tailored treatment strategies for SLE patients, as addressed in a review by Fasano et al. (48).

The use of non-invasive molecular imaging methods with specific markers will provide a more comprehensive picture of organ inflammation and enable serial assessments as patients are treated. The development of multimodality MRI scanners opens for high-resolution functional and molecular imaging research. Molecular imaging biomarkers will improve treatment to individual patients, and the capacity to evaluate the usefulness of new treatments in LN and other autoimmune diseases. Chronic inflammatory diseases like SLE are both debilitating and hard to diagnose. Future studies combining precise biomarkers, bioinformatical profiles and non-invasive imaging may lead to the development of procedures used for precise and early diagnosis, targeted treatment, as well as treatment and disease monitoring. This will increase the quality of life and life-expectancy for the patients.

## Author contributions

All authors listed have made a substantial, direct, and intellectual contribution to the work and approved it for publication.

## Funding

This research was funded in whole or in part by Northern Norway Regional Health Authority (HNF1343-17 and HNF1427-18), and UiT the Arctic University of Tromsø. For the purpose of Open Access, the author has applied a CC BY public copyright licence to any Author Accepted Manuscript (AAM) version arising from this submission.

## Conflict of interest

The authors declare that the research was conducted in the absence of any commercial or financial relationships that could be construed as a potential conflict of interest.

## Publisher’s note

All claims expressed in this article are solely those of the authors and do not necessarily represent those of their affiliated organizations, or those of the publisher, the editors and the reviewers. Any product that may be evaluated in this article, or claim that may be made by its manufacturer, is not guaranteed or endorsed by the publisher.

## Supplementary material

The Supplementary material for this article can be found online at: https://www.frontiersin.org/articles/10.3389/fmed.2023.1183535/full#supplementary-material

## Footnotes

^1^ https://www.3tr-imi.eu/

## References

[1] Furman D Campisi J Verdin E Carrera-Bastos P Targ S Franceschi C. Chronic inflammation in the etiology of disease across the life span. Nat Med. (2019) 25:1822–32. doi: 10.1038/s41591-019-0675-0, PMID: 31806905 PMC7147972

[2] Bieber K Hundt JE Yu X Ehlers M Petersen F Karsten CM. Autoimmune pre-disease. Autoimmun Rev. (2023) 22:103236. doi: 10.1016/j.autrev.2022.103236, PMID: 36436750

[3] Rekvig OP. The anti-DNA antibodies: their specificities for unique DNA structures and their unresolved clinical impact-a system criticism and a hypothesis. Front Immunol. (2021) 12:808008. doi: 10.3389/fimmu.2021.808008, PMID: 35087528 PMC8786728

[4] Giacomelli R Afeltra A Alunno A Bartoloni-Bocci E Berardicurti O Bombardieri M. Guidelines for biomarkers in autoimmune rheumatic diseases - evidence based analysis. Autoimmun Rev. (2019) 18:93–106. doi: 10.1016/j.autrev.2018.08.003, PMID: 30408582

[5] Nagafuchi Y Yanaoka H Fujio K. Lessons from transcriptome analysis of autoimmune diseases. Front Immunol. (2022) 13:857269. doi: 10.3389/fimmu.2022.857269, PMID: 35663941 PMC9157483

[6] Tang F Barbacioru C Wang Y Nordman E Lee C Xu N. mRNA-Seq whole-transcriptome analysis of a single cell. Nat Methods. (2009) 6:377–82. doi: 10.1038/nmeth.1315, PMID: 19349980

[7] Zeng L Yang K Zhang T Zhu X Hao W Chen H. Research progress of single-cell transcriptome sequencing in autoimmune diseases and autoinflammatory disease: a review. J Autoimmun. (2022) 133:102919. doi: 10.1016/j.jaut.2022.102919, PMID: 36242821

[8] Kuret T Sodin-Semrl S Leskosek B Ferk P. Single cell RNA sequencing in autoimmune inflammatory rheumatic diseases: current applications, challenges and a step toward precision medicine. Front Med (Lausanne). (2021) 8:822804. doi: 10.3389/fmed.2021.827095, PMID: 35118101 PMC8804286

[9] Penate Medina T Kolb JP Huttmann G Huber R Penate Medina O Ha L. Imaging inflammation - from whole body imaging to cellular resolution. Front Immunol. (2021) 12:692222. doi: 10.3389/fimmu.2021.692222, PMID: 34248987 PMC8264453

[10] Iking J Staniszewska M Kessler L Klose JM Luckerath K Fendler WP. Imaging inflammation with positron emission tomography. Biomedicine. (2021) 9:212. doi: 10.3390/biomedicines9020212, PMID: 33669804 PMC7922638

[11] Kang EH Ha YJ Lee YJ. Autoantibody biomarkers in rheumatic diseases. Int J Mol Sci. (2020) 21:1382. doi: 10.3390/ijms21041382, PMID: 32085664 PMC7073052

[12] Burbelo PD Iadarola MJ Keller JM Warner BM. Autoantibodies targeting intracellular and extracellular proteins in autoimmunity. Front Immunol. (2021) 12:548469. doi: 10.3389/fimmu.2021.548469, PMID: 33763057 PMC7982651

[13] Gomez-Banuelos E Fava A Andrade F. An update on autoantibodies in systemic lupus erythematosus. Curr Opin Rheumatol. (2023) 35:61–7. doi: 10.1097/BOR.0000000000000922, PMID: 36695053 PMC9881844

[14] Yu H Nagafuchi Y Fujio K. Clinical and immunological biomarkers for systemic lupus erythematosus. Biomol Ther. (2021) 11:928. doi: 10.3390/biom11070928, PMID: 34206696 PMC8301935

[15] Tan G Baby B Zhou Y Wu T. Emerging molecular markers towards potential diagnostic panels for lupus. Front Immunol. (2021) 12:808839. doi: 10.3389/fimmu.2021.808839, PMID: 35095896 PMC8792845

[16] Capecchi R Puxeddu I Pratesi F Migliorini P. New biomarkers in SLE: from bench to bedside. Rheumatology (Oxford). (2020) 59:v12–8. doi: 10.1093/rheumatology/keaa484, PMID: 32911542 PMC7719038

[17] Yu KY Yung SS Chau MK Tang CS Yap DY Tang AH. Clinico-pathological associations of serum VCAM-1 and ICAM-1 levels in patients with lupus nephritis. Lupus. (2021) 30:1039–50. doi: 10.1177/09612033211004727, PMID: 33765901

[18] Yu KYC Yung S Chau MKM Tang CSO Yap DYH Tang AHN. Serum syndecan-1, hyaluronan and thrombomodulin levels in patients with lupus nephritis. Rheumatology. (2021) 60:737–50. doi: 10.1093/rheumatology/keaa370, PMID: 32793966

[19] Genovese F Akhgar A Lim SS Farris AB Battle M Cobb J. Collagen type III and VI Remodeling biomarkers are associated with kidney fibrosis in lupus nephritis. Kidney360. (2021) 2:1473–81. doi: 10.34067/KID.0001132021, PMID: 35373114 PMC8786137

[20] He D Liu M Liu B. Interleukin-35 as a new biomarker of renal involvement in lupus nephritis patients. Tohoku J Exp Med. (2018) 244:263–70. doi: 10.1620/tjem.244.263, PMID: 29576585

[21] Wang M Huang L Peng LP Yang YM Mao J Zhu N. MALT1 serves as a biomarker for estimating disease risk of lupus nephritis: a prospective case-control study. Ann Transl Med. (2022) 10:3442. doi: 10.21037/atm-22-3442, PMID: 36111027 PMC9469135

[22] Idborg H Eketjall S Pettersson S Gustafsson JT Zickert A Kvarnstrom M. TNF-alpha and plasma albumin as biomarkers of disease activity in systemic lupus erythematosus. Lupus. Sci Med. (2018) 5:260. doi: 10.1136/lupus-2018-000260, PMID: 29955370 PMC6018889

[23] Fu Q He QY Dong Q Xie JY Geng YY Han H. The role of cyclic GMP-AMP synthase and interferon-I-inducible protein 16 as candidatebiomarkers of systemic lupus erythematosus. Clin Chim Acta. (2022) 524:69–77. doi: 10.1016/j.cca.2021.11.003, PMID: 34742679

[24] Xie YY Liu BY Wu ZW. Identification of serum biomarkers and pathways of systemic lupus erythematosus with skin involvement through GC/MS-based metabolomics analysis. Clin Cosmet Investig Dermatol. (2022) 15:77–86. doi: 10.2147/CCID.S345372, PMID: 35082507 PMC8784912

[25] Tumurkhuu G Csanova NG Kempf CL Laguna DE Camp SM Dagvadorj J. eNAMPT/TLR4 inflammatory cascade activation is a key contributor to SLE lung vasculitis and alveolar hemorrhage. J Transl Autoimmun. (2023) 6:100181. doi: 10.1016/j.jtauto.2022.100181, PMID: 36619655 PMC9816774

[26] Brilland B Bach-Bunner M Gomes CN Larochette V Foucher E Plaisance M. Serum Interleukin-26 is a new biomarker for disease activity assessment in systemic lupus erythematosus. Front Immunol. (2021) 12:663192. doi: 10.3389/fimmu.2021.663192, PMID: 34054830 PMC8160525

[27] Davies JC Midgley A Carlsson E Donohue S Bruce IN Beresford MW. Urine and serum S100A8/A9 and S100A12 associate with active lupus nephritis and may predict response to rituximab treatment. RMD Open. (2020) 6:1257. doi: 10.1136/rmdopen-2020-001257, PMID: 32723832 PMC7722276

[28] Lai X Xiang Y Zou L Li Y Zhang L. Elevation of serum CD5L concentration is correlated with disease activity in patients with systemic lupus erythematosus. Int Immunopharmacol. (2018) 63:311–6. doi: 10.1016/j.intimp.2018.07.022, PMID: 30173083

[29] Wang Y Huang Z Xiao Y Wan W Yang X. The shared biomarkers and pathways of systemic lupus erythematosus and metabolic syndrome analyzed by bioinformatics combining machine learning algorithm and single-cell sequencing analysis. Front Immunol. (2022) 13:1015882. doi: 10.3389/fimmu.2022.1015882, PMID: 36341378 PMC9627509

[30] Johnson NH Keane RW de Rivero Vaccari JP. Renal and inflammatory proteins as biomarkers of diabetic kidney disease and lupus nephritis. Oxidative Med Cell Longev. (2022) 2022:5631099. doi: 10.1155/2022/5631099, PMID: 35355862 PMC8958067

[31] Shipa M Santos LR Nguyen DX Embleton-Thirsk A Parvaz M Heptinstall LL. Identification of biomarkers to stratify response to B-cell-targeted therapies in systemic lupus erythematosus: an exploratory analysis of a randomised controlled trial. Lancet Rheumatol. (2023) 5:e24–35. doi: 10.1016/S2665-9913(22)00332-0, PMID: 36756239 PMC9894756

[32] Huang YJ Lin CH Yang HY Luo SF Kuo CF. Urine soluble CD163 is a promising biomarker for the diagnosis and evaluation of lupus nephritis. Front Immunol. (2022) 13:606. doi: 10.3389/fimmu.2022.1058606, PMID: 35911758 PMC9329951

[33] Fava A Rao DA Mohan C Zhang T Rosenberg A Fenaroli P. Urine proteomics and renal single-cell transcriptomics implicate Interleukin-16 in lupus nephritis. Arthritis Rheumatol. (2022) 74:829–39. doi: 10.1002/art.42023, PMID: 34783463 PMC9050800

[34] Hayry A Faustini F Zickert A Larsson A Niewold TB Svenungsson E. Interleukin (IL) 16: a candidate urinary biomarker for proliferative lupus nephritis. Lupus. Sci Med. (2022) 9:744. doi: 10.1136/lupus-2022-000744, PMID: 36104119 PMC9476119

[35] Perez-Arias AA Mendez-Perez RA Cruz C Zavala-Miranda MF Romero-Diaz J Marquez-Macedo SE. The first-year course of urine MCP-1 and its association with response to treatment and long-term kidney prognosis in lupus nephritis. Clin Rheumatol. (2023) 42:83–92. doi: 10.1007/s10067-022-06373-y, PMID: 36107264

[36] Gupta R Yadav A Aggarwal A. Longitudinal assessment of monocyte chemoattractant protein-1 in lupus nephritis as a biomarker of disease activity. Clin Rheumatol. (2016) 35:2707–14. doi: 10.1007/s10067-016-3404-9, PMID: 27624649

[37] Ju W Nair V Smith S Zhu L Shedden K Song PXK. Tissue transcriptome-driven identification of epidermal growth factor as a chronic kidney disease biomarker. Sci Transl Med. (2015) 7:316ra193. doi: 10.1126/scitranslmed.aac7071, PMID: 26631632 PMC4861144

[38] Azukaitis K Ju W Kirchner M Nair V Smith M Fang Z. Low levels of urinary epidermal growth factor predict chronic kidney disease progression in children. Kidney Int. (2019) 96:214–21. doi: 10.1016/j.kint.2019.01.035, PMID: 31005273

[39] Martinez-Rojas MA Sanchez-Navarro A Mejia-Vilet JM Perez-Villalva R Uribe N Bobadilla NA. Urinary serpin-A3 is an early predictor of clinical response to therapy in patients with proliferative lupus nephritis. Am J Physiol Renal Fluid Electrol Physiol. (2022) 323:F425–34. doi: 10.1152/ajprenal.00099.2022, PMID: 35834275

[40] Bertolo M Baumgart S Durek P Peddinghaus A Mei H Rose T. Deep phenotyping of urinary leukocytes by mass cytometry reveals a leukocyte signature for early and non-invasive prediction of response to treatment in active lupus nephritis. Front Immunol. (2020) 11:256. doi: 10.3389/fimmu.2020.00256, PMID: 32265898 PMC7105605

[41] Lee EJ Kwon OC Ghang B Lim DH Kim DH Hong S. Immunoglobulin binding protein 1 as a potential urine biomarker in patients with lupus nephritis. Int J Mol Sci. (2019) 20:2606. doi: 10.3390/ijms20102606, PMID: 31137925 PMC6567280

[42] Sun F Teng J Yu P Li W Chang J Xu H. Involvement of TWEAK and the NF-kappaB signaling pathway in lupus nephritis. Exp Ther Med. (2018) 15:2611–9. doi: 10.3892/etm.2018.5711, PMID: 29456665 PMC5795405

[43] Doron R Merav L Nasrin E Adi SD Elias T Gleb S. Low urine secretion of Semaphorin3A in lupus patients with proteinuria. Inflammation. (2022) 45:603–9. doi: 10.1007/s10753-021-01570-4, PMID: 34562225

[44] Burcsar S Toldi G Kovacs L Szalay B Vasarhelyi B Balog A. Urine soluble urokinase plasminogen activator receptor as a potential biomarker of lupus nephritis activity. Biomarkers. (2021) 26:443–9. doi: 10.1080/1354750X.2021.1910343, PMID: 33825610

[45] Davies JC Carlsson E Midgley A Smith EMD Bruce IN Beresford MW. A panel of urinary proteins predicts active lupus nephritis and response to rituximab treatment. Rheumatology (Oxford). (2021) 60:3747–59. doi: 10.1093/rheumatology/keaa851, PMID: 33313921 PMC8328509

[46] Mok CC Soliman S Ho LY Mohamed FA Mohamed FI Mohan C. Urinary angiostatin, CXCL4 and VCAM-1 as biomarkers of lupus nephritis. Arthritis Res Ther. (2018) 20:6. doi: 10.1186/s13075-017-1498-3, PMID: 29325582 PMC5765646

[47] Perez-Hernandez J Martinez-Arroyo O Ortega A Galera M Solis-Salguero MA Chaves FJ. Urinary exosomal miR-146a as a marker of albuminuria, activity changes and disease flares in lupus nephritis. J Nephrol. (2021) 34:1157–67. doi: 10.1007/s40620-020-00832-y, PMID: 32803682

[48] Fasano S Milone A Nicoletti GF Isenberg DA Ciccia F. Precision medicine in systemic lupus erythematosus. Nat Rev Rheumatol. (2023) 19:331–42. doi: 10.1038/s41584-023-00948-y, PMID: 37041269

[49] Newman-Rivera AM Kurzhagen JT Rabb H. TCR??+CD4-/CD8-?Double negative? T cells in health and disease-implications for the kidney. Kidney Int. (2022) 102:25–37. doi: 10.1016/j.kint.2022.02.035, PMID: 35413379 PMC9233047

[50] Wu Z Zheng Y Sheng J Han Y Yang Y Pan H. CD3(+)CD4(−)CD8(−) (double-negative) T cells in inflammation, Immune Disorders and Cancer. Front Immunol. (2022) 13:816005. doi: 10.3389/fimmu.2022.1008047, PMID: 35222392 PMC8866817

[51] Velikkakam T Gollob KJ Dutra WO. Double-negative T cells: setting the stage for disease control or progression. Immunology. (2022) 165:371–85. doi: 10.1111/imm.13441, PMID: 34939192 PMC10626195

[52] Alexander JJ Jacob A Chang A Quigg RJ Jarvis JN. Double negative T cells, a potential biomarker for systemic lupus erythematosus. Precis Clin Med. (2020) 3:34–43. doi: 10.1093/pcmedi/pbaa001, PMID: 32257532 PMC7093895

[53] Fang Y Ni J Wang YS Zhao Y Jiang LQ Chen C. Exosomes as biomarkers and therapeutic delivery for autoimmune diseases: opportunities and challenges. Autoimmun Rev. (2023) 22:3260. doi: 10.1016/j.autrev.2022.103260, PMID: 36565798

[54] Perez-Hernandez J Forner MJ Pinto C Chaves FJ Cortes R Redon J. Increased urinary Exosomal MicroRNAs in patients with systemic lupus erythematosus. PLoS One. (2015) 10:e0138618. doi: 10.1371/journal.pone.0138618, PMID: 26390437 PMC4577109

[55] Lee JY Park JK Lee EY Lee EB Song YW. Circulating exosomes from patients with systemic lupus erythematosus induce an proinflammatory immune response. Arthritis Res Ther. (2016) 18:264. doi: 10.1186/s13075-016-1159-y, PMID: 27852323 PMC5112700

[56] Wang W Yue C Gao S Li S Zhou J Chen J. Promising roles of Exosomal microRNAs in systemic lupus erythematosus. Front Immunol. (2021) 12:757096. doi: 10.3389/fimmu.2021.757096, PMID: 34966383 PMC8710456

[57] Wu H Chen S Li A Shen K Wang S Wang S. LncRNA expression profiles in systemic lupus erythematosus and rheumatoid arthritis: emerging biomarkers and therapeutic targets. Front Immunol. (2021) 12:792884. doi: 10.3389/fimmu.2021.792884, PMID: 35003113 PMC8732359

[58] Pang Y Tan Y Li YZ Zhang JC Guo YB Guo ZL. Pentraxin 3 is closely associated with tubulointerstitial injury in lupus nephritis a large Multicenter cross-sectional study. Medicine. (2016) 95:2520. doi: 10.1097/MD.0000000000002520, PMID: 26817892 PMC4998266

[59] Jiang Z Shao M Dai X Pan Z Liu D. Identification of diagnostic biomarkers in systemic lupus erythematosus based on bioinformatics analysis and machine learning. Front Genet. (2022) 13:865559. doi: 10.3389/fgene.2022.1061550, PMID: 35495164 PMC9047905

[60] Hofmann SC Bosma A Bruckner-Tuderman L Vukmanovic-Stejic M Jury EC Isenberg DA. Invariant natural killer T cells are enriched at the site of cutaneous inflammation in lupus erythematosus. J Dermatol Sci. (2013) 71:22–8. doi: 10.1016/j.jdermsci.2013.04.012, PMID: 23664188

[61] Skiljevic D Bonaci-Nikolic B Brasanac D Nikolic M. Apoptosis of keratinocytes and serum DNase I activity in patients with cutaneous lupus erythematosus: relationship with clinical and immunoserological parameters. J Eur Acad Dermatol. (2017) 31:523–9. doi: 10.1111/jdv.13943, PMID: 27557471

[62] Rolla G Brussino L Bertero MT Colagrande P Converso M Bucca C. Increased nitric oxide in exhaled air of patients with systemic lupus erythematosus. J Rheumatol. (1997) 24:1066–71. PMID: 9195510

[63] Brunner AM Wander SA Neuberg D Sadrzadeh H Ballen KK Amrein PC. Diagnostic features and 2-hydroxyglutarate (2-HG) levels among acute myeloid Leukemia (AML) patients with and without isocitrate dehydrogenase (IDH) mutations. Blood. (2014) 124:1045. doi: 10.1182/blood.V124.21.1045.1045

[64] Li YQ Wang DY Li Y Zhu JL Zhao JL Deng YC. A highly sensitive Sandwich ELISA to detect CSF progranulin: a potential biomarker for CNS disorders. J Neuropath Exp Neur. (2019) 78:406–15. doi: 10.1093/jnen/nlz022, PMID: 30939191

[65] Chen WS Lin KC Chen CH Liao HT Wang HP Li WY. Autoantibody and biopsy grading are associated with expression of ICAM-1, MMP-3, and TRAIL in salivary gland mononuclear cells of Chinese patients with Sjogren's syndrome. J Rheumatol. (2009) 36:989–96. doi: 10.3899/jrheum.080733, PMID: 19332626

[66] Mao ZM Tan Y Tao J Li LL Wang H Yu F. Renal mTORC1 activation is associated with disease activity and prognosis in lupus nephritis. Rheumatology. (2022) 61:3830–40. doi: 10.1093/rheumatology/keac037, PMID: 35040950 PMC9608003

[67] Pedersen HL Horvei KD Thiyagarajan D Norby GE Seredkina N Moroni G. Lupus nephritis: low urinary DNase I levels reflect loss of renal DNase I and may be utilized as a biomarker of disease progression. J Pathol Clin Res. (2018) 4:193–203. doi: 10.1002/cjp2.99, PMID: 29624903 PMC6065113

[68] Bouachi K Moktefi A Zhang SY Oniszczuk J Sendeyo K Remy P. Expression of CMIP in podocytes is restricted to specific classes of lupus nephritis. PLoS One. (2018) 13:7066. doi: 10.1371/journal.pone.0207066, PMID: 30439969 PMC6237342

[69] Becker AM Dao KH Han BK Kornu R Lakhanpal S Mobley AB. SLE peripheral blood B cell, T cell and myeloid cell transcriptomes display unique profiles and each subset contributes to the interferon signature. PLoS One. (2013) 8:e67003. doi: 10.1371/journal.pone.0067003, PMID: 23826184 PMC3691135

[70] Wardowska A Komorniczak M Skoniecka A Bullo-Piontecka B Lisowska KA Debska-Slizien MA. Alterations in peripheral blood B cells in systemic lupus erythematosus patients with renal insufficiency. Int Immunopharmacol. (2020) 83:106451. doi: 10.1016/j.intimp.2020.106451, PMID: 32248020

[71] Nehar-Belaid D Hong S Marches R Chen G Bolisetty M Baisch J. Mapping systemic lupus erythematosus heterogeneity at the single-cell level. Nat Immunol. (2020) 21:1094–106. doi: 10.1038/s41590-020-0743-0, PMID: 32747814 PMC7442743

[72] Trzupek D Lee M Hamey F Wicker LS Todd JA Ferreira RC. Single-cell multi-omics analysis reveals IFN-driven alterations in T lymphocytes and natural killer cells in systemic lupus erythematosus. Wellcome Open Res. (2021) 6:149. doi: 10.12688/wellcomeopenres.16883.1, PMID: 35509371 PMC9046903

[73] Ashton MP Eugster A Dietz S Loebel D Lindner A Kuehn D. Association of Dendritic Cell Signatures with Autoimmune Inflammation Revealed by single-cell profiling. Arthritis Rheumatol. (2019) 71:817–28. doi: 10.1002/art.40793, PMID: 30511817

[74] Zhang R Li Y Pan B Li Y Liu A Li X. Increased expression of hub gene CXCL10 in peripheral blood mononuclear cells of patients with systemic lupus erythematosus. Exp Ther Med. (2019) 18:4067–75. doi: 10.3892/etm.2019.8013, PMID: 31616519 PMC6781829

[75] Tang Y Li X Wei Y Sun Y Yang Y Zhang X. A preliminary study of KAT2A on cGAS-related immunity in inflammation amplification of systemic lupus erythematosus. Cell Death Dis. (2021) 12:1036. doi: 10.1038/s41419-021-04323-1, PMID: 34718330 PMC8557211

[76] Perez RK Gordon MG Subramaniam M Kim MC Hartoularos GC Targ S. Single-cell RNA-seq reveals cell type-specific molecular and genetic associations to lupus. Science. (2022) 376:eabf1970. doi: 10.1126/science.abf1970, PMID: 35389781 PMC9297655

[77] Li H Boulougoura A Endo Y Tsokos GC. Abnormalities of T cells in systemic lupus erythematosus: new insights in pathogenesis and therapeutic strategies. J Autoimmun. (2022) 132:102870. doi: 10.1016/j.jaut.2022.102870, PMID: 35872102

[78] Pan HF Leng RX Feng CC Li XP Chen GM Li BZ. Expression profiles of Th17 pathway related genes in human systemic lupus erythematosus. Mol Biol Rep. (2013) 40:391–9. doi: 10.1007/s11033-012-2073-2, PMID: 23054011

[79] Ronnblom L Leonard D. Interferon pathway in SLE: one key to unlocking the mystery of the disease. Lupus Sci Med. (2019) 6:e000270. doi: 10.1136/lupus-2018-000270, PMID: 31497305 PMC6703304

[80] Buang N Tapeng L Gray V Sardini A Whilding C Lightstone L. Type I interferons affect the metabolic fitness of CD8(+) T cells from patients with systemic lupus erythematosus. Nat Commun. (2021) 12:1980. doi: 10.1038/s41467-021-22312-y, PMID: 33790300 PMC8012390

[81] Yin X Kim K Suetsugu H Bang SY Wen L Koido M. Biological insights into systemic lupus erythematosus through an immune cell-specific transcriptome-wide association study. Ann Rheum Dis. (2022) 81:1273–80. doi: 10.1136/annrheumdis-2022-222345, PMID: 35609976 PMC9380500

[82] Tangtanatakul P Thammasate B Jacquet A Reantragoon R Pisitkun T Avihingsanon Y. Transcriptomic profiling in human mesangial cells using patient-derived lupus autoantibodies identified miR-10a as a potential regulator of IL8. Sci Rep. (2017) 7:14517. doi: 10.1038/s41598-017-15160-8, PMID: 29109423 PMC5673966

[83] dos Santos M Bringhenti RN Rodrigues PG do Nascimento JF Pereira SV Zancan R. Podocyte-associated mRNA profiles in kidney tissue and in urine of patients with active lupus nephritis. Int J Clin Exp Pathol. (2015) 8:4600–13. PMID: 26191151 PMC4503023

[84] Der E Suryawanshi H Morozov P Kustagi M Goilav B Ranabothu S. Tubular cell and keratinocyte single-cell transcriptomics applied to lupus nephritis reveal type I IFN and fibrosis relevant pathways. Nat Immunol. (2019) 20:915–27. doi: 10.1038/s41590-019-0386-1, PMID: 31110316 PMC6584054

[85] Arazi A Rao DA Berthier CC Davidson A Liu Y Hoover PJ. The immune cell landscape in kidneys of patients with lupus nephritis. Nat Immunol. (2019) 20:902–14. doi: 10.1038/s41590-019-0398-x, PMID: 31209404 PMC6726437

[86] Gilmore AC Wilson HR Cairns TD Botto M Lightstone L Bruce IN. Immune gene expression and functional networks in distinct lupus nephritis classes. Lupus Sci Med. (2022) 9:615. doi: 10.1136/lupus-2021-000615, PMID: 35074933 PMC8788334

[87] Dunlap GS Billi AC Xing X Ma F Maz MP Tsoi LC. Single-cell transcriptomics reveals distinct effector profiles of infiltrating T cells in lupus skin and kidney. JCI. Insight. (2022) 7:341. doi: 10.1172/jci.insight.156341, PMID: 35290245 PMC9089784

[88] Crickx E Tamirou F Huscenot T Costedoat-Chalumeau N Rabant M Karras A. Molecular signatures of kidney antibody-secreting cells in lupus patients with active nephritis upon immunosuppressive therapy. Arthritis Rheumatol. (2021) 73:1461–6. doi: 10.1002/art.41703, PMID: 33645886

[89] Parikh SV Malvar A Song H Shapiro J Mejia-Vilet JM Ayoub I. Molecular profiling of kidney compartments from serial biopsies differentiate treatment responders from non-responders in lupus nephritis. Kidney Int. (2022) 102:845–65. doi: 10.1016/j.kint.2022.05.033, PMID: 35788359 PMC9613357

[90] Tsoi LC Hile GA Berthier CC Sarkar MK Reed TJ Liu J. Hypersensitive IFN responses in lupus keratinocytes reveal key mechanistic determinants in cutaneous lupus. J Immunol. (2019) 202:2121–30. doi: 10.4049/jimmunol.1800650, PMID: 30745462 PMC6424612

[91] Szeto CC Kwan BC Tam LS. Urinary mRNA in systemic lupus erythematosus. Adv Clin Chem. (2013) 62:197–219. doi: 10.1016/B978-0-12-800096-0.00005-6 24772668

[92] Akhgar A Sinibaldi D Zeng L Farris AB 3rd Cobb J Battle M. Urinary markers differentially associate with kidney inflammatory activity and chronicity measures in patients with lupus nephritis. Lupus Sci Med. (2023) 10:747. doi: 10.1136/lupus-2022-000747, PMID: 36717181 PMC9887703

[93] Billi AC Ma F Plazyo O Gharaee-Kermani M Wasikowski R Hile GA. Nonlesional lupus skin contributes to inflammatory education of myeloid cells and primes for cutaneous inflammation. Sci Transl Med. (2022) 14:eabn2263. doi: 10.1126/scitranslmed.abn2263, PMID: 35476593 PMC9169615

[94] Maier-Moore JS Koelsch KA Smith K Lessard CJ Radfar L Lewis D. Antibody-secreting cell specificity in labial salivary glands reflects the clinical presentation and serology in patients with Sjogren's syndrome. Arthritis Rheumatol. (2014) 66:3445–56. doi: 10.1002/art.38872, PMID: 25199908 PMC4245382

[95] Guo C Liu Q Zong D Zhang W Zuo Z Yu Q. Single-cell transcriptome profiling and chromatin accessibility reveal an exhausted regulatory CD4+ T cell subset in systemic lupus erythematosus. Cell Rep. (2022) 41:111606. doi: 10.1016/j.celrep.2022.111606, PMID: 36351407

[96] Zheng M Hu Z Mei X Ouyang L Song Y Zhou W. Single-cell sequencing shows cellular heterogeneity of cutaneous lesions in lupus erythematosus. Nat Commun. (2022) 13:7489. doi: 10.1038/s41467-022-35209-1, PMID: 36470882 PMC9722937

[97] Li Y Ma C Liao S Qi S Meng S Cai W. Combined proteomics and single cell RNA-sequencing analysis to identify biomarkers of disease diagnosis and disease exacerbation for systemic lupus erythematosus. Front Immunol. (2022) 13:969509. doi: 10.3389/fimmu.2022.1072573, PMID: 36524113 PMC9746895

[98] Itotagawa E Tomofuji Y Kato Y Konaka H Tsujimoto K Park J. SLE stratification based on BAFF and IFN-I bioactivity for biologics and implications of BAFF produced by glomeruli in lupus nephritis. Rheumatology (Oxford). (2022) 62:1988–97. doi: 10.1093/rheumatology/keac528 PMC10152287 36094336

[99] Banchereau R Hong S Cantarel B Baldwin N Baisch J Edens M. Personalized Immunomonitoring uncovers molecular networks that stratify lupus patients. Cells. (2016) 165:551–65. doi: 10.1016/j.cell.2016.03.008, PMID: 27040498 PMC5426482

[100] Ishigaki K Shoda H Kochi Y Yasui T Kadono Y Tanaka S. Quantitative and qualitative characterization of expanded CD4+ T cell clones in rheumatoid arthritis patients. Sci Rep. (2015) 5:12937. doi: 10.1038/srep12937, PMID: 26245356 PMC4542667

[101] Tuller T Atar S Ruppin E Gurevich M Achiron A. Common and specific signatures of gene expression and protein-protein interactions in autoimmune diseases. Genes Immun. (2013) 14:67–82. doi: 10.1038/gene.2012.55, PMID: 23190644

[102] Liao L Liang K Lan L Wang J Guo J. Marker genes change of synovial fibroblasts in rheumatoid arthritis patients. Biomed Res Int. (2021) 2021:5544264. doi: 10.1155/2021/5544264, PMID: 34195267 PMC8203351

[103] Zerrouk N Miagoux Q Dispot A Elati M Niarakis A. Identification of putative master regulators in rheumatoid arthritis synovial fibroblasts using gene expression data and network inference. Sci Rep. (2020) 10:16236. doi: 10.1038/s41598-020-73147-4, PMID: 33004899 PMC7529794

[104] Stephenson W Donlin LT Butler A Rozo C Bracken B Rashidfarrokhi A. Single-cell RNA-seq of rheumatoid arthritis synovial tissue using low-cost microfluidic instrumentation. Nat Commun. (2018) 9:791. doi: 10.1038/s41467-017-02659-x, PMID: 29476078 PMC5824814

[105] Zhang F Wei K Slowikowski K Fonseka CY Rao DA Kelly S. Defining inflammatory cell states in rheumatoid arthritis joint synovial tissues by integrating single-cell transcriptomics and mass cytometry. Nat Immunol. (2019) 20:928–42. doi: 10.1038/s41590-019-0378-1, PMID: 31061532 PMC6602051

[106] Simmons DP Nguyen HN Gomez-Rivas E Jeong Y Jonsson AH Chen AF. SLAMF7 engagement superactivates macrophages in acute and chronic inflammation. Sci Immunol. (2022) 7:eabf2846. doi: 10.1126/sciimmunol.abf2846, PMID: 35148199 PMC8991457

[107] Li X Sun H Li H Li D Cai Z Xu J. A single-cell RNA-sequencing analysis of distinct subsets of synovial macrophages in rheumatoid arthritis. DNA Cell Biol. (2023) 42:212–22. doi: 10.1089/dna.2022.0509, PMID: 36940312

[108] Yamada S Nagafuchi Y Wang M Ota M Hatano H Takeshima Y. Immunomics analysis of rheumatoid arthritis identified precursor dendritic cells as a key cell subset of treatment resistance. Ann Rheum Dis. (2023) 82:809–19. doi: 10.1136/ard-2022-223645, PMID: 36918189 PMC10314026

[109] Argyriou A Wadsworth MH Lendvai A Christensen SM Hensvold AH Gerstner C. Single cell sequencing identifies clonally expanded synovial CD4(+) T(PH) cells expressing GPR56 in rheumatoid arthritis. Nat Commun. (2022) 13:4046. doi: 10.1038/s41467-022-31519-6, PMID: 35831277 PMC9279430

[110] Moon JS Younis S Ramadoss NS Iyer R Sheth K Sharpe O. Cytotoxic CD8(+) T cells target citrullinated antigens in rheumatoid arthritis. Nat Commun. (2023) 14:319. doi: 10.1038/s41467-022-35264-8, PMID: 36658110 PMC9852471

[111] Wampler Muskardin TL Fan W Jin Z Jensen MA Dorschner JM Ghodke-Puranik Y. Distinct single cell gene expression in peripheral blood monocytes correlates with tumor necrosis factor inhibitor treatment response groups defined by type I interferon in rheumatoid arthritis. Front Immunol. (2020) 11:1384. doi: 10.3389/fimmu.2020.01384, PMID: 32765497 PMC7378891

[112] Murthy S Karkossa I Schmidt C Hoffmann A Hagemann T Rothe K. Danger signal extracellular calcium initiates differentiation of monocytes into SPP1/osteopontin-producing macrophages. Cell Death Dis. (2022) 13:53. doi: 10.1038/s41419-022-04507-3, PMID: 35022393 PMC8755842

[113] Orange DE Yao V Sawicka K Fak J Frank MO Parveen S. RNA identification of PRIME cells predicting rheumatoid arthritis flares. N Engl J Med. (2020) 383:218–28. doi: 10.1056/NEJMoa2004114, PMID: 32668112 PMC7546156

[114] Micheroli R Elhai M Edalat S Frank-Bertoncelj M Burki K Ciurea A. Role of synovial fibroblast subsets across synovial pathotypes in rheumatoid arthritis: a deconvolution analysis. RMD Open. (2022) 8:e001949. doi: 10.1136/rmdopen-2021-001949, PMID: 34987094 PMC8734041

[115] Yang J Zhang Y Liang J Yang X Liu L Zhao H. Fibronectin-1 is a dominant mechanism for rheumatoid arthritis via the mediation of synovial fibroblasts activity. Front Cell Dev Biol. (2022) 10:1010114. doi: 10.3389/fcell.2022.1010114, PMID: 36225320 PMC9548557

[116] Gao A Zhao W Wu R Su R Jin R Luo J. Tissue-resident memory T cells: the key frontier in local synovitis memory of rheumatoid arthritis. J Autoimmun. (2022) 133:102950. doi: 10.1016/j.jaut.2022.102950, PMID: 36356551

[117] Ramesh A Schubert RD Greenfield AL Dandekar R Loudermilk R Sabatino JJ Jr. A pathogenic and clonally expanded B cell transcriptome in active multiple sclerosis. Proc Natl Acad Sci U S A. (2020) 117:22932–43. doi: 10.1073/pnas.2008523117, PMID: 32859762 PMC7502747

[118] van der Poel M Ulas T Mizee MR Hsiao CC Miedema SSM Adelia. Transcriptional profiling of human microglia reveals grey-white matter heterogeneity and multiple sclerosis-associated changes. Nat Commun. (2019) 10:1139. doi: 10.1038/s41467-019-08976-7, PMID: 30867424 PMC6416318

[119] Liu J Gao H Li C Zhu F Wang M Xu Y. Expression and regulatory characteristics of peripheral blood immune cells in primary Sjogren's syndrome patients using single-cell transcriptomic. iScience. (2022) 25:105509. doi: 10.1016/j.isci.2022.105509, PMID: 36425764 PMC9678742

[120] He Y Chen R Zhang M Wang B Liao Z Shi G. Abnormal changes of monocyte subsets in patients with Sjogren's syndrome. Front Immunol. (2022) 13:864920. doi: 10.3389/fimmu.2022.1093990, PMID: 35309355 PMC8931697

[121] Hou X Hong X Ou M Meng S Wang T Liao S. Analysis of gene expression and TCR/B cell receptor profiling of immune cells in primary Sjogren's syndrome by single-cell sequencing. J Immunol. (2022) 209:238–49. doi: 10.4049/jimmunol.2100803, PMID: 35705251

[122] Xu T Zhu HX You X Ma JF Li X Luo PY. Single-cell profiling reveals pathogenic role and differentiation trajectory of granzyme K+CD8+ T cells in primary Sjogren's syndrome. JCI Insight. (2023) 8:490. doi: 10.1172/jci.insight.167490, PMID: 36881472 PMC10243796

[123] Hong X Meng S Tang D Wang T Ding L Yu H. Single-cell RNA sequencing reveals the expansion of cytotoxic CD4(+) T lymphocytes and a landscape of immune cells in primary Sjogren's syndrome. Front Immunol. (2020) 11:594658. doi: 10.3389/fimmu.2020.594658, PMID: 33603736 PMC7884617

[124] Mandric I Schwarz T Majumdar A Hou K Briscoe L Perez R. Optimized design of single-cell RNA sequencing experiments for cell-type-specific eQTL analysis. Nat Commun. (2020) 11:5504. doi: 10.1038/s41467-020-19365-w, PMID: 33127880 PMC7599215

[125] Ma Y Chen J Wang T Zhang L Xu X Qiu Y. Accurate machine learning model to diagnose chronic autoimmune diseases utilizing information from B cells and monocytes. Front Immunol. (2022) 13:870531. doi: 10.3389/fimmu.2022.1044462, PMID: 35515003 PMC9065417

[126] Stahl PL Salmen F Vickovic S Lundmark A Navarro JF Magnusson J. Visualization and analysis of gene expression in tissue sections by spatial transcriptomics. Science. (2016) 353:78–82. doi: 10.1126/science.aaf2403, PMID: 27365449

[127] Carlberg K Korotkova M Larsson L Catrina AI Stahl PL Malmstrom V. Exploring inflammatory signatures in arthritic joint biopsies with spatial transcriptomics. Sci Rep. (2019) 9:18975. doi: 10.1038/s41598-019-55441-y, PMID: 31831833 PMC6908624

[128] Hardt U Carlberg K Af Klint E Sahlstrom P Larsson L van Vollenhoven A. Integrated single cell and spatial transcriptomics reveal autoreactive differentiated B cells in joints of early rheumatoid arthritis. Sci Rep. (2022) 12:11876. doi: 10.1038/s41598-022-15293-5, PMID: 35831338 PMC9279471

[129] Vickovic S Schapiro D Carlberg K Lotstedt B Larsson L Hildebrandt F. Three-dimensional spatial transcriptomics uncovers cell type localizations in the human rheumatoid arthritis synovium. Commun Biol. (2022) 5:129. doi: 10.1038/s42003-022-03050-3, PMID: 35149753 PMC8837632

[130] Kaufmann M Evans H Schaupp AL Engler JB Kaur G Willing A. Identifying CNS-colonizing T cells as potential therapeutic targets to prevent progression of multiple sclerosis. Med (N Y). (2021) 2:296–312 e8. doi: 10.1016/j.medj.2021.01.006, PMID: 33748804 PMC7966680

[131] Misrielal C Alsema AM Wijering MHC Miedema A Mauthe M Reggiori F. Transcriptomic changes in autophagy-related genes are inversely correlated with inflammation and are associated with multiple sclerosis lesion pathology. Brain Behav Immun Health. (2022) 25:100510. doi: 10.1016/j.bbih.2022.100510, PMID: 36120103 PMC9478930

[132] Wu H Liu F Shangguan Y Yang Y Shi W Hu W. Integrating spatial transcriptomics with single-cell transcriptomics reveals a spatiotemporal gene landscape of the human developing kidney. Cell Biosci. (2022) 12:80. doi: 10.1186/s13578-022-00801-x, PMID: 35659756 PMC9164720

[133] Salem F Perin L Sedrakyan S Angeletti A Ghiggeri GM Coccia MC. The spatially resolved transcriptional profile of acute T cell-mediated rejection in a kidney allograft. Kidney Int. (2022) 101:131–6. doi: 10.1016/j.kint.2021.09.004, PMID: 34555393 PMC9387544

[134] Bell RMB Denby L. Myeloid heterogeneity in kidney disease as revealed through single-cell RNA sequencing. Kidney360. (2021) 2:1844–51. doi: 10.34067/KID.0003682021, PMID: 35372996 PMC8785845

[135] Cheung MD Agarwal A George JF. Where are they now: spatial and molecular diversity of tissue-resident macrophages in the kidney. Semin Nephrol. (2022) 42:151276. doi: 10.1016/j.semnephrol.2022.10.002, PMID: 36435683

[136] Fritz D Inamo J Zhang F. Single-cell computational machine learning approaches to immune-mediated inflammatory disease: new tools uncover novel fibroblast and macrophage interactions driving pathogenesis. Front Immunol. (2022) 13:1076700. doi: 10.3389/fimmu.2022.1076700, PMID: 36685542 PMC9846263

[137] Noel T Wang QS Greka A Marshall JL. Principles of spatial transcriptomics analysis: a practical walk-through in kidney tissue. Front Physiol. (2021) 12:809346. doi: 10.3389/fphys.2021.809346, PMID: 35069263 PMC8770822

[138] Zheng Z Chang L Li J Wu Y Chen G Zou L. Insights gained and future outlook from scRNAseq studies in autoimmune rheumatic diseases. Front Immunol. (2022) 13:849050. doi: 10.3389/fimmu.2022.1095657, PMID: 35251048 PMC8891165

[139] Madissoon E Oliver AJ Kleshchevnikov V Wilbrey-Clark A Polanski K Richoz N. A spatially resolved atlas of the human lung characterizes a gland-associated immune niche. Nat Genet. (2023) 55:66–77. doi: 10.1038/s41588-022-01243-4, PMID: 36543915 PMC9839452

[140] Chiricosta L Blando S D'Angiolini S Gugliandolo A Mazzon E. A comprehensive exploration of the transcriptomic landscape in multiple sclerosis: a systematic review. Int J Mol Sci. (2023) 24:1448. doi: 10.3390/ijms24021448, PMID: 36674968 PMC9862618

[141] Lin WY Lan JL Wang SJ. Gallium-67 scintigraphy to predict response to therapy in active lupus nephritis. J Nucl Med. (1998) 39:2137–41. PMID: 9867157

[142] Hsieh TY Lin YC Hung WT Chen YM Wen MC Chen HH. Change of renal gallium uptake correlated with change of inflammation activity in renal pathology in lupus nephritis patients. J Clin Med. (2021) 10:4654. doi: 10.3390/jcm10204654, PMID: 34682781 PMC8541120

[143] Thurman JM Serkova NJ. Non-invasive imaging to monitor lupus nephritis and neuropsychiatric systemic lupus erythematosus. F1000Res. (2015) 4:153. doi: 10.12688/f1000research.6587.2, PMID: 26309728 PMC4536614

[144] Mauro D Barbagallo G D’Angelo S Sannino P Naty S Bruno C. Role of positron emission tomography for central nervous system involvement in systemic autoimmune diseases: status and perspectives. Curr Med Chem. (2018) 25:3096–104. doi: 10.2174/0929867324666170523144402, PMID: 28545366

[145] Rapacchi S Smith RX Wang Y Yan L Sigalov V Krasileva KE. Towards the identification of multi-parametric quantitative MRI biomarkers in lupus nephritis. Magn Reson Imaging. (2015) 33:1066–74. doi: 10.1016/j.mri.2015.06.019, PMID: 26119419

[146] Zheng Z Wang Y Yan T Jia J Li D Wei L. Detection of renal hypoxia configuration in patients with lupus nephritis: a primary study using blood oxygen level-dependent MR imaging. Abdom Radiol (NY). (2021) 46:2032–44. doi: 10.1007/s00261-020-02794-y, PMID: 33079255

[147] Shi H Jia J Li D Wei L Shang W Zheng Z. Blood oxygen level dependent magnetic resonance imaging for detecting pathological patterns in lupus nephritis patients: a preliminary study using a decision tree model. BMC Nephrol. (2018) 19:33. doi: 10.1186/s12882-017-0787-z, PMID: 29426280 PMC5806290

[148] Zheng Z Yan T Jia J Li D Wei L Shang W. Assessment of renal pathological changes in lupus nephritis using diffusion weighted imaging: a multiple correspondence analysis. Kidney Blood Press Res. (2018) 43:847–59. doi: 10.1159/000490333, PMID: 29870992

[149] Shi H Yan T Li D Jia J Shang W Wei L. Detection of renal hypoxia in lupus nephritis using blood oxygen level-dependent MR imaging: a multiple correspondence analysis. Kidney Blood Press Res. (2017) 42:123–35. doi: 10.1159/000472720, PMID: 28437782

[150] Li X Xu X Zhang Q Ren H Zhang W Liu Y. Diffusion weighted imaging and blood oxygen level-dependent MR imaging of kidneys in patients with lupus nephritis. J Transl Med. (2014) 12:295. doi: 10.1186/s12967-014-0295-x, PMID: 25342208 PMC4221678

[151] Chen YX Zhou W Ye YQ Zeng L Wu XF Ke B. Clinical study on the use of advanced magnetic resonance imaging in lupus nephritis. BMC Med Imaging. (2022) 22:210. doi: 10.1186/s12880-022-00928-w, PMID: 36451131 PMC9713986

[152] Wang L Xiong C Li M Zeng X Wang Q Fang W. Assessment of lung glucose uptake in patients with systemic lupus erythematosus pulmonary arterial hypertension: a quantitative FDG-PET imaging study. Ann Nucl Med. (2020) 34:407–14. doi: 10.1007/s12149-020-01461-y, PMID: 32314147

[153] Makis W Ciarallo A Gonzalez-Verdecia M Probst S. Systemic lupus erythematosus associated pitfalls on (18)F-FDG PET/CT: reactive follicular hyperplasia, Kikuchi-Fujimoto disease, inflammation and lymphoid hyperplasia of the spleen mimicking lymphoma. Nucl Med Mol Imaging. (2018) 52:74–9. doi: 10.1007/s13139-017-0471-z, PMID: 29391916 PMC5777954

[154] Curiel R Akin EA Beaulieu G DePalma L Hashefi M. PET/CT imaging in systemic lupus erythematosus. Ann N Y Acad Sci. (2011) 1228:71–80. doi: 10.1111/j.1749-6632.2011.06076.x 21718325

[155] Nwaubani P Cercignani M Colasanti A. In vivo quantitative imaging of hippocampal inflammation in autoimmune neuroinflammatory conditions: a systematic review. Clin Exp Immunol. (2022) 210:24–38. doi: 10.1093/cei/uxac058, PMID: 35802780 PMC9585553

[156] Carlucci PM Purmalek MM Dey AK Temesgen-Oyelakin Y Sakhardande S Joshi AA. Neutrophil subsets and their gene signature associate with vascular inflammation and coronary atherosclerosis in lupus. JCI Insight. (2018) 3:276. doi: 10.1172/jci.insight.99276, PMID: 29669944 PMC5931124

[157] Shin JI Lee KH Park S Yang JW Kim HJ Song K. Systemic lupus erythematosus and lung involvement: a comprehensive review. J Clin Med. (2022) 11:6714. doi: 10.3390/jcm11226714, PMID: 36431192 PMC9698564

[158] De Feo MS Pontico M Frantellizzi V Corica F De Cristofaro F De Vincentis G. Zr-89-PET imaging in humans: a systematic review. Clin Transl Imaging. (2022) 10:23–36. doi: 10.1007/s40336-021-00462-9

[159] Clausen AS Christensen C Christensen E Cold S Kristensen LK Hansen AE. Development of a cu-64-labeled CD4(+) T cell targeting PET tracer: evaluation of CD4 specificity and its potential use in collagen-induced arthritis. EJNMMI Res. (2022) 12:934. doi: 10.1186/s13550-022-00934-7, PMID: 36114433 PMC9481863

[160] van der Krogt JMA van Binsbergen WH van der Laken CJ Tas SW. Novel positron emission tomography tracers for imaging of rheumatoid arthritis. Autoimmun Rev. (2021) 20:102764. doi: 10.1016/j.autrev.2021.102764, PMID: 33476822

[161] Palazzo L Lindblom J Mohan C Parodis I. Current insights on biomarkers in lupus nephritis: a systematic review of the literature. J Clin Med. (2022) 11:759. doi: 10.3390/jcm11195759, PMID: 36233628 PMC9570701

[162] Zhang Y Lee TY. Revealing the immune heterogeneity between systemic lupus erythematosus and rheumatoid arthritis based on multi-omics data analysis. Int J Mol Sci. (2022) 23:5166. doi: 10.3390/ijms23095166, PMID: 35563556 PMC9101622

