# Supplementary material for: Advanced methods and novel biomarkers in autoimmune diseases ‑ a review of the recent years progress in systemic lupus erythematosus
Source: Front Med (Lausanne). 2023 Jun 23;10:1183535. doi: 10.3389/fmed.2023.1183535 (PMC10326284; doi:10.3389/fmed.2023.1183535)
Supplement: Supplementary file 1 [file Table_1.docx]

| SUPplementary Table 1 | Number of papers found within the different fields | | | | | | |
| --- | --- | --- | --- | --- | --- | --- | --- |
|  | **Inflammation AND "organ" NOT virus NOT animal NOT cancer** | | | | | | |
| Diseases | **Brain** | **Joint** | **Kidney** | **Lung** | **Pancreas** | **Salivary Gland** | **Skin** |
| SLE | 123 | 294 | 527 | 150 | 8 | 27 | 387 |
| RA | 229 | 6045 | 245 | 383 | 16 | 346 | 516 |
| MS | 1659 | 54 | 44 | 59 | 5 | 2 | 97 |
| SS | 21 | 71 | 35 | 61 | 9 | 394 | 40 |
| DT1 | 41 | 22 | 152 | 20 | 210 | 12 | 49 |

| SUPplementary Table 2. | Number of papers found within the different fields | | | | |
| --- | --- | --- | --- | --- | --- |
|  | NOT virus NOT animal NOT cancer | | | | |
| Diseases | **Biomarker** | **Transcriptomics** | **scRNAseq** | **Imaging** | **Molecular imaging** |
| SLE | 5250 | 514 | 347 | 4664 | 107 |
| RA | 9012 | 911 | 438 | 14883 | 360 |
| MS | 5700 | 545 | 334 | 16614 | 688 |
| SS | 1207 | 112 | 44 | 1536 | 29 |
| T1D | 4658 | 274 | 266 | 3270 | 102 |

| SUPplementary Table 3. | | Number of papers found within the different fields | | | | | |
| --- | --- | --- | --- | --- | --- | --- | --- |
|  | **Inflammation and “field”** **NOT virus NOT animal NOT cancer** | | | | | |  |
| Diseases | **Biomarker** | | **Transcriptomics** | **scRNAseq** | **Imaging** | **Molecular imaging** |  |
| SLE | 689 | | 95 | 29 | 357 | 4 |  |
| RA | 2075 | | 263 | 83 | 1858 | 66 |  |
| MS | 810 | | 93 | 35 | 1099 | 14 |  |
| SS | 174 | | 19 | 6 | 99 | 5 |  |
| T1D | 366 | | 37 | 24 | 91 | 2 |  |
| LN | 186 | | 22 | 7 | 34 | 1 |  |

| SUPplementary TABLE 4. | | Number of papers found within the different fields | | | | |
| --- | --- | --- | --- | --- | --- | --- |
| Inflammation and.. NOT virus NOT animal NOT cancer | | | | | | |
|  | **Biomarker** | | **Transcriptomics** | **scRNAseq** | **Imaging** | **Molecular imaging** |
| SLE kidney | 126 | | 17 | 4 | 26 | 2 |
| RA joint | 949 | | 134 | 30 | 1213 | 13 |
| MS brain | 307 | | 34 | 14 | 775 | 11 |
| SS SG | 69 | | 10 | 4 | 38 | 3 |
| T1D pancreas | 35 | | 16 | 3 | 19 | 2 |

| SUPplementary Table 5. |  |  |  |  |  |
| --- | --- | --- | --- | --- | --- |
| Organs | **Biomarker** | **Transcriptomics** | **scRNAseq** | **Imaging** | **Molecular imaging** |
| Brain | 137 | 6 | 4 | 1062 | 29 |
| Joint | 184 | 30 | 15 | 436 | 16 |
| Kidney | 762 | 66 | 21 | 311 | 13 |
| Liver | 72 | 4 | 6 | 90 | 5 |
| Lung | 97 | 7 | 6 | 387 | 8 |
| Pancreas | 7 | 1 | 1 | 10 | 0 |
| Peripheral blood | 694 | 148 | 76 | 81 | 6 |
| Salivary gland | 19 | 1 | 1 | 36 | 0 |
| Skin | 246 | 42 | 18 | 203 | 7 |

| SUPplementary Table 6. | Number of papers found within the different fields | | | | | | | |
| --- | --- | --- | --- | --- | --- | --- | --- | --- |
| Organs | **Transcriptomics** | **Transcriptomics and inflammation** | | | **scRNAseq** | **Single cell sequencing and Inflammation** | | |
| Brain | 7 | | 0 | | 5 | | | 1 |
| Joint | 33 | | 9 | 17 | | | 2 | |
| Kidney | 75 | | 19 | | 25 | | | 4 |
| Liver | 6 | | 1 | 6 | | | 1 | |
| Lung | 14 | | 3 | | 7 | | | 0 |
| Pancreas | 1 | | 1 | 1 | | | 1 | |
| Peripheral blood | 170 | | 29 | | 82 | | | 4 |
| Salivary gland | 2 | | 0 | 0 | | | 0 | |
| Skin | 47 | | 16 | | 20 | | | 2 |
| Urine | 17 | | 7 | | 5 | | | 3 |

| SUPplementary Table 7. | Number of papers found within the different fields | | |
| --- | --- | --- | --- |
| Disease | **scRNAseq** | **Inflammation and scRNAseq** | **Spatial transcriptomics** |
| RA | 24 | 13 | 8 |
| MS | 14 | 4 | 6 |
| SS | 6 | 1 | 2 |
| T1D | 10 | 1 | 0 |

| SUPplementary Table 8. | Number of papers found within the different fields | | | | |
| --- | --- | --- | --- | --- | --- |
| Organs | **Imaging** | **Inflammation and Imaging** | **Molecular Imaging** | **MRI** | **PET** |
| Brain | 1010 | 59 | 27 | 838 | 48 |
| Joint | 419 | 48 | 15 | 147 | 4 |
| Kidney | 287 | 26 | 12 | 78 | 9 |
| Liver | 80 | 3 | 4 | 24 | 0 |
| Lung | 351 | 26 | 7 | 24 | 7 |
| Pancreas | 8 | 1 | 0 | 4 | 0 |
| Peripheral blood | 75 | 0 | 5 | 21 | 5 |
| Salivary gland | 34 | 5 | 0 | 6 | 1 |
| Skin | 183 | 34 | 5 | 54 | 6 |
